# Kinematically distinct saccades are used in a context-dependent manner by larval zebrafish

**DOI:** 10.1016/j.cub.2024.08.008

**Published:** 2024-09-04

**Authors:** Charles K. Dowell, Joanna Y.N. Lau, Paride Antinucci, Isaac H. Bianco

**Affiliations:** 1 Department of Neuroscience, Physiology & Pharmacology, UCL, Gower Street, London WC1E 6BT, UK

## Abstract

Saccades are rapid eye movements that are used by all species with good vision. In this study, we set out to characterize the complete repertoire of larval zebrafish horizontal saccades to gain insight into their contributions to visually guided behavior and underlying neural control. We identified five saccade types, defined by systematic differences in kinematics and binocular coordination, which were differentially expressed across a variety of behavioral contexts. Conjugate saccades formed a large group that serves at least four functions. These include fast phases of the optokinetic nystagmus, visual scanning in stationary animals, and shifting gaze in coordination with body turns. In addition, we discovered a previously undescribed pattern of eye-body coordination in which small conjugate saccades partially oppose head rotation to maintain gaze during forward locomotion. Convergent saccades were coordinated with body movements to foveate prey targets during hunting. Detailed kinematic analysis showed that conjugate and convergent saccades differed in the millisecond coordination of the eyes and body and followed distinct velocity main sequence relationships. This challenges the prevailing view that all horizontal saccades are controlled by a common brainstem circuit and instead indicates saccade-type-specific neural control.

## Introduction

Saccades are brief but extremely rapid eye movements that are observed across species and phyla from crabs and cuttlefish to mice and primates.^1^ They function to quickly shift the direction of gaze between fixations and intermittently recenter the eyes during compensatory nystagmus (vestibuloocular and optokinetic reflexes [OKRs]). Most species coordinate saccades with head rotations; however, they are also used independently of head movements in foveate animals to successively shift the point of fixation for high-spatial frequency sampling of the visual environment.^2^ Much is known about the kinematics of saccades and their underlying neurophysiology.^3,4^ Although latency, duration, and velocity can vary as a function of whether saccades are made when the head is free to move versus restrained,^5^ in the dark versus light,^6^ or directed to visible versus remembered targets,^7^ it is generally considered that all saccades follow the same kinematic rules and are generated by a common brainstem circuit. It has been argued that saccades provide an optimal visual sampling strategy and that the timing and location of fixations reflect the allocation of attention during specific cognitive tasks.^8,9^ Moreover, studies during naturalistic human behavior have indicated that saccades are under top-down control, pre-dominantly directing foveal gaze to task-relevant objects in a manner that is shaped by learned internal models of the world and which serves to reduce uncertainly about high-priority environmental variables.^10,11^ Thus, saccadic eye movements can provide important insights into a species’ visuomotor strategies and cognitive processes.

The larval zebrafish is an important model in neuroscience research^12^ and has been used to study the development and neural control of oculomotor behaviors. Most studies of saccades have focused on spontaneous conjugate saccades and fast phases of the OKR in restrained animals and have revealed neural activity that controls the timing and direction of spontaneous saccades,^13,14^ optogenetically mapped the locus of saccade generation to rhombomere 5,^15^ and described monocular and binocular encoding in saccade-active cells.^16^ In addition, studies of prey-catching behavior have shown that zebrafish initiate hunting with a convergent saccade and high ocular vergence is then sustained throughout prey tracking.^17,18^ However, the extent to which these encompass the diversity of saccadic eye movements is unknown, and there has been little examination of saccade kinematics.

Here, we set out to describe the full repertoire of saccades of larval zebrafish by measuring rapid eye movements in tethered and freely swimming animals engaged in a range of visuomotor behaviors. We distinguished five saccade types, defined by systematic differences in kinematics and binocular coordination, and found that they were differentially engaged across behavioral contexts. Conjugate saccades formed a large group with four identifiable visual roles, including two opposing patterns of coordination with head/body rotations during turns versus forward locomotion. Convergent saccades were coordinated with turns during hunting to produce target-directed gaze shifts to foveate prey. High-temporal-resolution recordings revealed that conjugate and convergent saccades differed in the millisecond timing of binocular eye movements and eye-body coordination, and remarkably, conformed to different velocity main sequence relationships, indicating that they are controlled by distinct patterns of neural activity. Overall, this study provides insight into the visuomotor behavioral strategies used by larval zebrafish and motivates hypotheses about circuit control of saccades and eye-body coordination.

## Results

### Characterization of the saccadic repertoire of larval zebrafish

**Figure 1:**
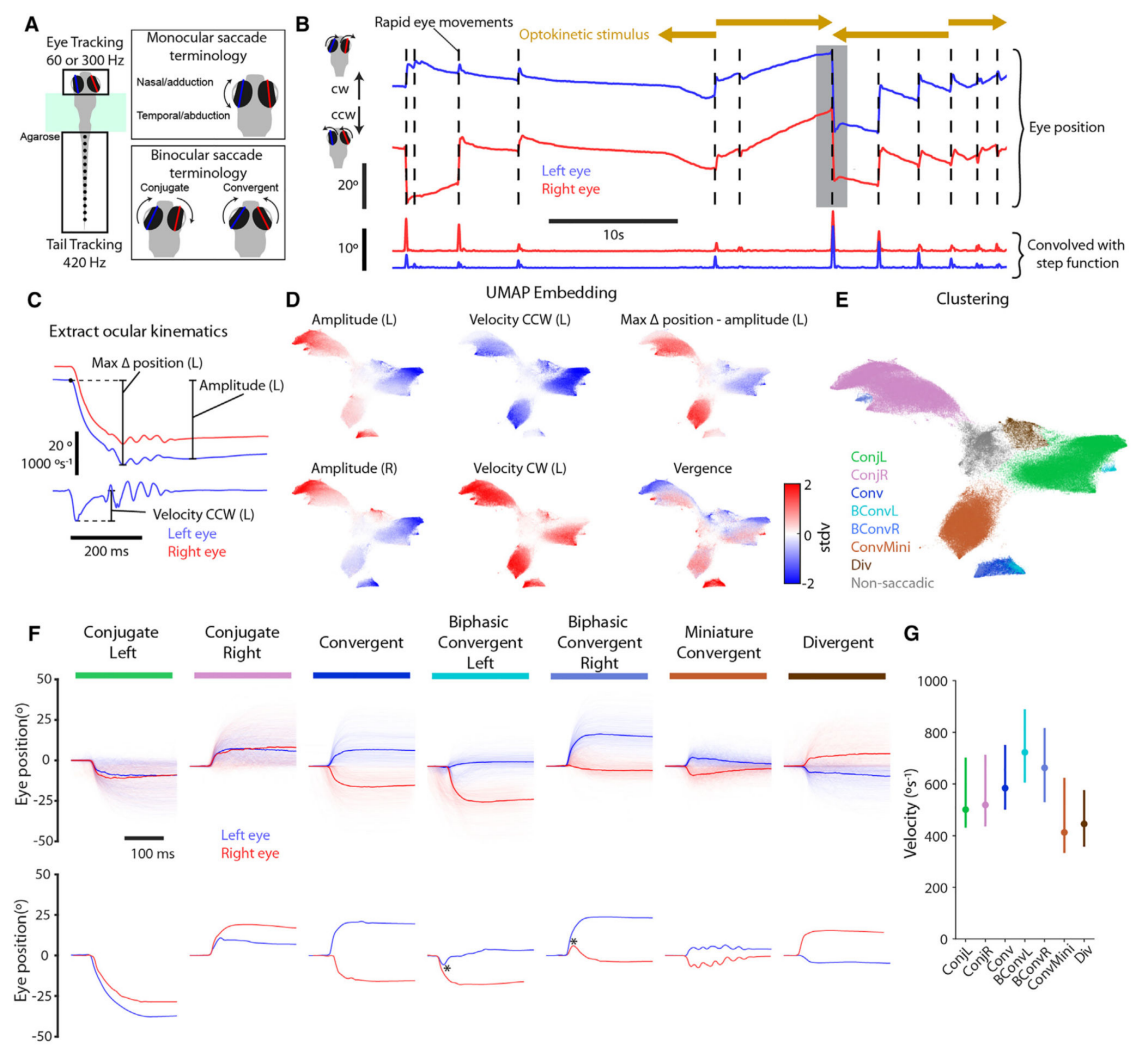
Saccade detection and classification (A) Illustration of tethered behavioral tracking and key to saccade terminology. (B) Eye position time series data from an example 60 s experimental epoch. Upward deflections correspond to rightward rotation of both eyes (i.e., left eye nasal and right eye temporal). Lower plot shows convolution of eye position with a step function; rapid eye movements were detected as peaks in this trace, indicated with dashed lines. During this epoch, drifting gratings were presented in front of the animal to evoke optokinetic response, as indicated. (C) A subset of position (top) and velocity (bottom) metrics for an example rapid eye movement (time of this event indicated by gray shading in B). Letters in parentheses indicate left or right eye. See STAR Methods for details. (D) Rapid eye movements (213,462 events from 152 animals) embedded in 2D UMAP space and colored by normalized oculomotor metrics. Additional metrics shown in Figure S1. (E) Same UMAP embedding colored by saccade type label. (F) Top: for each saccade type, 500 eye position traces are plotted with the median overlaid in bold. Bottom: single example saccades. Asterisk (*) indicates reversal of eye velocity during biphasic convergent saccades. (G) Eye velocity for all saccade types. Data plotted as median (IQR) across mean values from each animal (*n* = 152). See also Figures S1 and S2.

To estimate the full repertoire of saccadic eye movements of larval zebrafish, we tracked the behavior of both freely swimming and tethered animals engaged in a range of behaviors. Tethered larvae (Figure 1A) were presented with visual stimuli that included whole-field drifting gratings, which evoke optokinetic nystagmus (OKR, comprising slow phase rotations in the direction of visual motion with intermittent fast “reset” saccades)^19^ and optomotor swimming,^20,21^ small, prey-like moving spots that evoke hunting responses including convergent saccades,^17^ as well as dark flashes and looming stimuli that evoke high-angle turns and avoidance swims.^22,23^ Freely swimming larvae were allowed to explore an arena and hunt live prey.

We described the kinematic features of rapid eye movements, initially focusing on datasets from tethered animals where we could obtain very high-quality tracking data (*n* = 152 larvae, 6–7 days post fertilization). Putative rapid eye movements were first detected as peaks in the convolution of the eye-in-head position (hereafter eye position) time series and a step filter (Figure 1B). For each event, we then computed nine position and velocity metrics (Figure 1C; STAR Methods). We note that putative saccadic events were characterized in terms of movement of both eyes to enable assessment of different patterns of binocular coordination. Kinematics included the amplitude and peak velocity of each eye’s movement, post-saccadic vergence, and a metric describing the extent to which eye position was maintained after the putative saccade (the difference between max delta position and amplitude). Next, we used uniform manifold approximation and projection (UMAP) to embed *n* = 213,462 events in two dimensions (2D) and observed a smooth and systematic variation of kinematic values across the embedding space (Figures 1D and S1A). Several regions appeared to be well separated from one another and contained rapid eye movements with similar kinematic distributions. We therefore applied a density-based clustering procedure to the embedded data and thereby classified rapid eye movements using seven cluster labels (Figure 1E). Clusters had distinct and unimodal kinematic distributions (Figures S1B–S1D), indicating that this classification scheme captured the major patterns of variation across the dataset. We note that hierarchical clustering applied to the non-embedded data (and where the number of clusters was not directly specified), produced a similar description of saccade space (data not shown).

The seven labels defined five saccade types (two types were subdivided into left- and right-directed clusters), which included both conjugate and disjunctive eye rotations, wherein the left and right eyes moved either in the same or opposite directions, respectively (Figure 1F). Conjugate saccades were assigned to two large clusters (left- and right-directed “Conj”), within which there was continuous variation in kinematic properties (Figures 1D and S1). Convergent saccades, in which both eyes rotate nasally, fell into four clusters: regular convergent saccades (“Conv”) and biphasic convergent saccades (left- and right-directed “BConv”) both resulted in large and sustained elevations in vergence (Figure S1D); biphasic saccades had the distinctive feature that one eye first made a small temporal rotation before reversing direction and rotating nasally (Figure 1F). Zebrafish also generated a large number of miniature convergent saccades (“ConvMini”) involving a small, transient increase in vergence that decayed rapidly. Finally, we observed a small number of divergent saccades (“Div”). Eye velocity varied systematically across saccade types, with median values ranging from 400 to over 700°/s (Figure 1G).

We could identify the same saccade types in freely swimming animals. To show this, we extracted the same nine kinematics and transformed the data into the previously learned low-dimensional embedding space (*n* = 9,367 events from eight animals). Despite the fact that this was a smaller dataset, rapid eye movements from freely swimming larvae spanned the embedding space and could be classified with the same seven labels (Figure S2A). Inspection of eye position traces confirmed that these saccades showed similar features, including patterns of binocular coordination, compared with those of tethered animals (Figure S2B).

### Saccade types show context-dependent use

Saccadic eye movements serve a variety of visual functions. Therefore, we next investigated how the saccade types of larval zebrafish are utilized across different behavioral contexts.

**Figure 2:**
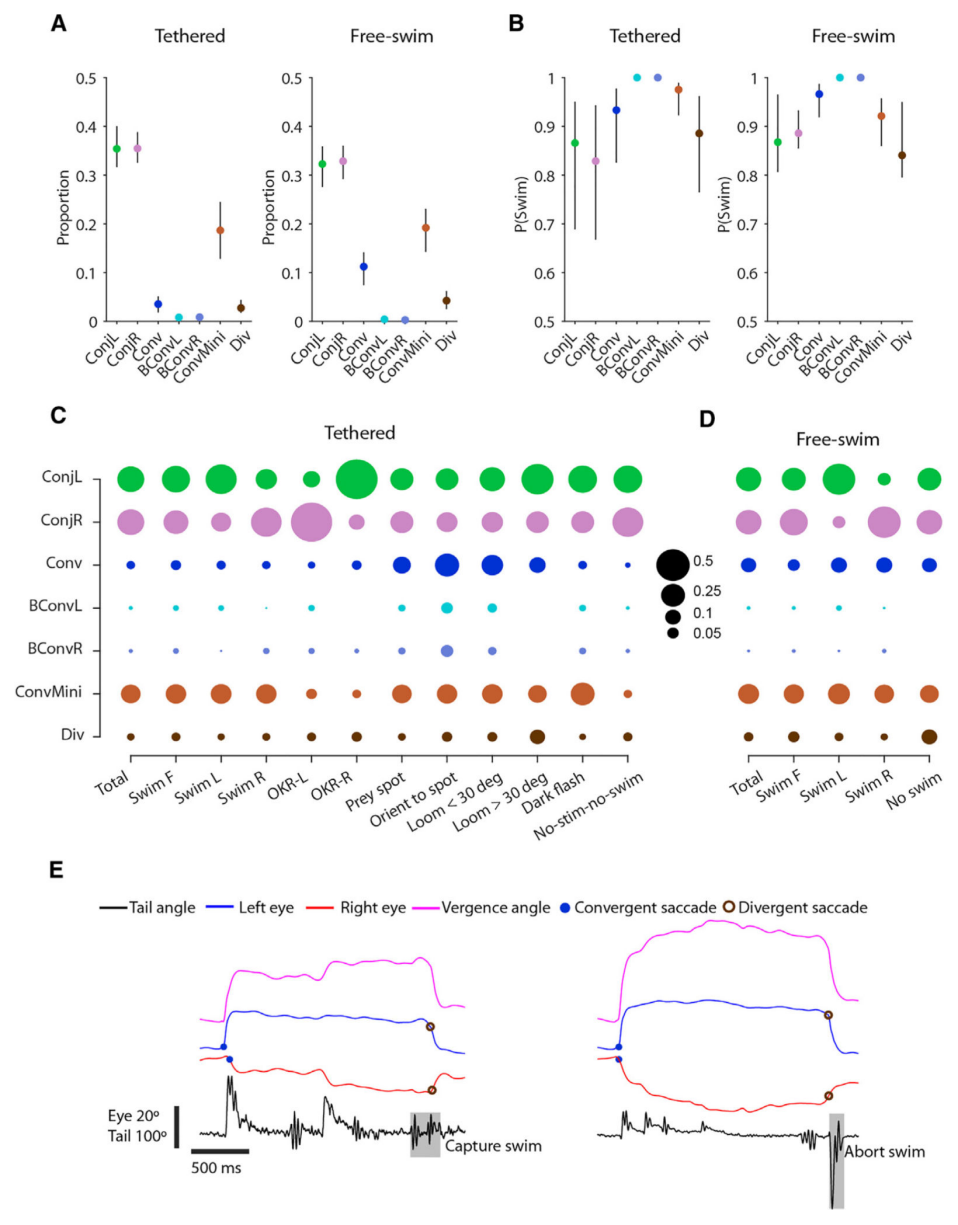
Contextual deployment of saccades (A) Proportion of each saccade type for tethered and free-swimming datasets (median and IQR across *n* = 152 tethered and *n* = 8 free-swimming larvae). (B) Probability of a swim occurring within 200 ms of a saccade, for each saccade type. (C) Proportion of saccades corresponding to each type during the indicated contexts, for tethered larvae. Swim F, forward swim; Swim R/L, right/left swim (abs. tail bend angle ≥ 25°); OKR-L/R, left/rightward OKR grating and no swim; prey spot, small prey-like moving spot; orient to spot, first orienting turn to prey-like stimulus. (D) Proportion of saccades corresponding to each type during the indicated contexts, for free-swimming larvae. Swim F, forward swim; Swim R/L, right/left swim (abs. orientation change ≥10°). (E) Examples of hunting sequences that end with a divergent saccade coincident with either a capture swim (left) or an abort swim (right). See also Tables S1, S2, and S3.

Conjugate saccades were the most frequent type of rapid eye movement (~70% of all saccades in both tethered and freely swimming animals; Figure 2A; Tables S1, S2, and S3). As was the case for all saccade types, they were usually accompanied by a swim bout (Figure 2B) but were also produced by stationary animals (Figures 2C and 2D). When either tethered or freely swimming animals generated left or right turns (“Swim L/R”), we observed an elevated frequency of conjugate saccades of the same laterality, suggesting that they contribute to coordinated eye-body gaze shifts, in line with previous observations.^14^ When presented with drifting gratings to evoke the OKR, larvae often generated conjugate saccades, unaccompanied by swims, in the opposite direction to whole-field motion, indicating that these are fast phases that function to recenter the eye in the orbit (Figure 2C).

Convergent saccades are a defining feature of hunting,^17,18^ and in accordance with previous studies, we observed an elevated frequency of both regular and biphasic convergent saccades when larvae were presented with, and oriented toward, small prey-like moving spots (Figure 2C). Convergent saccades also occurred “spontaneously,” at a low rate,^24,25^ and were quite frequently evoked by looming stimuli, especially while the expanding spot was still small (<30°) and presumably perceived as a prey-like stimulus.

Miniature convergent saccades were observed in most contexts (Figures 2C and 2D), making their role rather unclear. However, they were rarely observed in tethered, stationary larvae. Divergent saccades were uncommon but occurred at elevated frequency in response to looming stimuli (>30°), compatible with a role in redirecting gaze behind the animal in combination with the high-angle avoidance turns elicited by this stimulus.^23,26^ They also occur at the end of hunting sequences to switch out of the high-vergence predatory mode of gaze; thus, we observed divergent saccades after larvae performed capture strikes or aborted prey tracking (Figure 2E).

Because conjugate (Conj) and convergent (Conv and BConv) saccades spanned a similar range of amplitudes but showed substantial variation in their utilization across behavioral contexts, we focused on these types for the remainder of the study. In particular, we characterized how these saccades are coordinated with swims to generate gaze shifts and compared their detailed kinematics to make inferences about underlying circuit activity.

### Conjugate saccades are coordinated with swims for gaze shifting and gaze maintenance

Across many species, planned gaze shifts are accomplished by coordinated eye and head movements, but in some species, saccades alone can redirect gaze when the head/body is stationary.^2^ Because conjugate saccades in zebrafish occurred both with and without accompanying swims, we next explored the ways in which they contribute to gaze changes.

**Figure 3:**
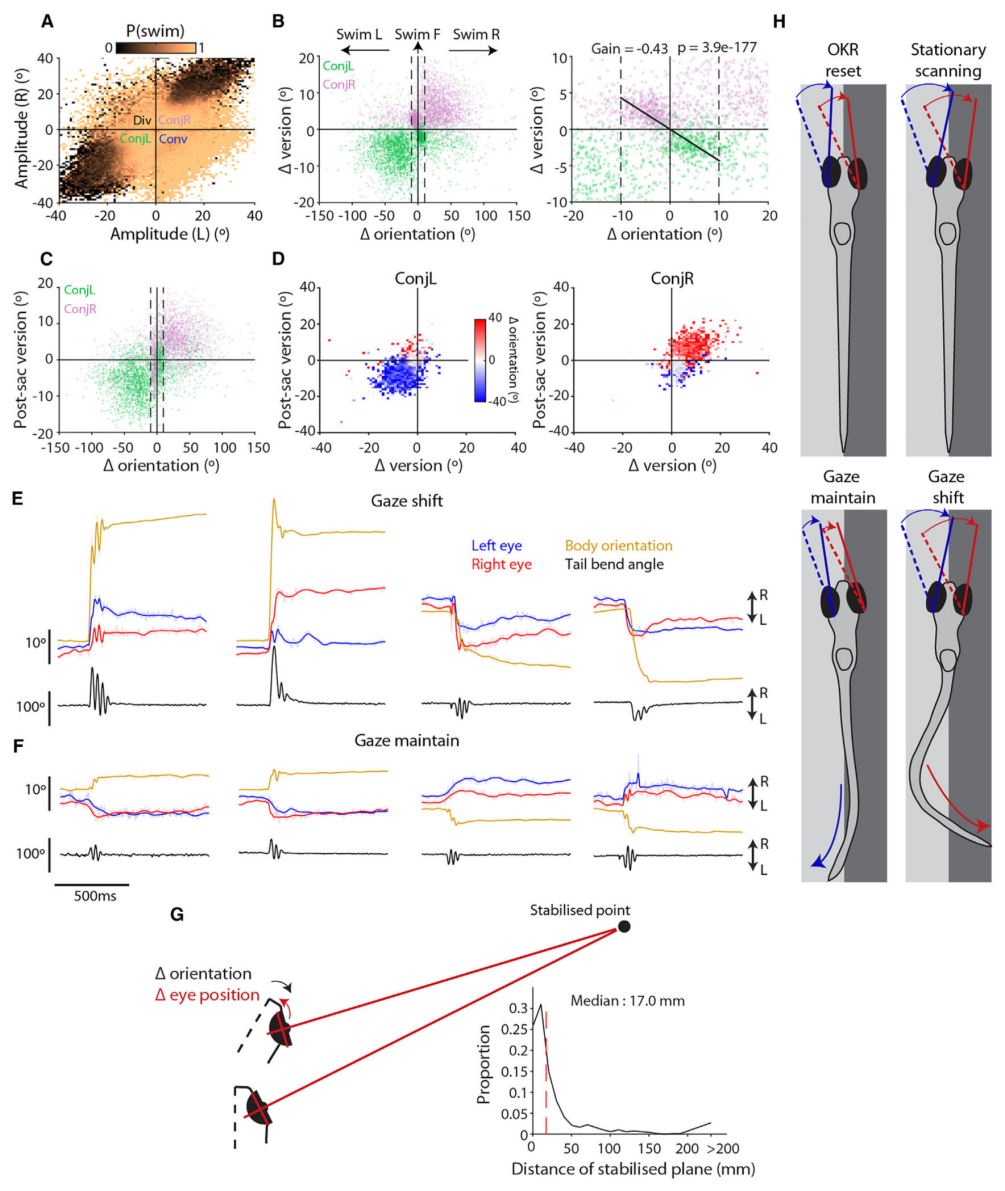
Conjugate saccades are produced by stationary animals and are coordinated with swims to either shift or maintain gaze (A) Saccade amplitude coded by the probability of a coincident swim (data from 152 tethered larvae). (B) Left: ocular version change (mean of left- and right-eye amplitude) versus change in body orientation (5,869 saccades from 8 free-swimming fish). Dashed lines indicate thresholds for forward swims (−10 ≤ Δori ≤ 10°) versus turns. Right: magnified portion highlighting gaze-maintaining conjugate saccades. Linear fit calculated for saccades with direction opposite to body reorientation and where |Δori| < 10° (gradient = 0.43, *R*^2^ = 0.39, *n* = 820 saccades, *p* value, t test). (C) Post-saccadic version (mean of left- and right-eye position) versus change in body orientation. (D) Left and right conjugate saccades binned by change in version and post-saccadic version and color-coded by median change in body orientation. (E and F) Examples of gaze-shifting (E) and gaze-maintaining (F) conjugate saccades. Smoothed eye position is plotted in bold over raw data. (G) Left: schematic illustrating that gaze-maintain saccades serve to stabilize a visual plane at a finite distance from the animal during locomotion. Further detail in Figure S3. Right: distribution of distances of stabilized point for 664 gaze-maintain saccade-swim events. (H) Summary of four contexts in which larval zebrafish use conjugate saccades. See also Figure S3.

Zebrafish generated their largest amplitude conjugate saccades when they were stationary. This was evident for both “spontaneous” conjugate saccades and OKR fast phases (i.e., those not accompanied by tail motion; Figures S1E–S1H). Furthermore, a 2D histogram of saccade amplitude coded by the probability of a coincident swim (defined as swim bouts within 200 ms of saccade onset) showed that, in tethered animals, conjugate saccades exceeding 20° were rarely accompanied by body movement (Figure 3A). This relationship was also observed under freely swimming conditions (Figure S3A), albeit at lower frequency because larvae that were free to move spent little time stationary. These observations indicate that zebrafish can redirect gaze using saccadic eye movements alone and that when they are stationary, large amplitude conjugate saccades allow them to maintain visual exploration and scan their environment.

Next, we examined the kinematics of saccades that were coincident with swims and observed two distinct relationships between eye and body reorientations. First, we observed that left- and right-directed turns were usually accompanied by conjugate saccades that shifted gaze in the same direction (Figure 2). To investigate this in more detail, we plotted the change in body orientation of freely swimming larvae versus the change in version produced by the saccadic eye movement (version describes conjugate eye position and is the arithmetic mean of left- and right-eye positions). This showed that for body orientation changes exceeding 10°, the majority (86.2% [80.1%−88.5%], median interquartile range [IQR], *n* = 5,869) were accompanied by conjugate saccades of the same laterality (Figure 3B). Although the change in version was weakly correlated with body rotation (Pearson’s rho = 0.09, *p* = 2.1 × 10^9^) (Figure 3B, upper right and lower left quadrants) and the overall gaze shift (rho = 0.20, *p* = 2.2 × 10^37^) (Figure S3C), eye position following the gaze shift was consistently displaced in the direction of locomotion (Figure 3C). Specifically, post-saccadic version matched turn direction for 88.7% (81.7%−92.2%) of gaze shifts (for which absolute change in body orientation exceeded 10°). We also showed this by color-coding post-saccadic version by body reorientation (Figure 3D) (or swim lateralization for tethered animals; Figure S3B) and found that rightward conjugate saccades that terminated rightward of primary eye position were associated with rightward swims. To summarize, zebrafish shift their gaze using combined eye-body movements and orient their visual field in the direction in which they are moving (Figure 3E).

We also discovered that zebrafish have a second, unexpected pattern of eye-body coordination, in which conjugate saccades are paired with swims of the opposite laterality. For instance, rightward conjugate saccades were sometimes coincident with leftward body movements. This was especially evident when changes in body orientation were small, <10°, within a range that can be considered as “forward swims”^27^ (Figure 3B, right). The fact that these eye movements were saccades, as opposed to oscillations produced by compensatory spino-ocular coupling,^28^ was evidenced by both raw eye tracking data (Figure 3F), as well as analysis of velocity main sequence relationships (see below). The magnitude of the change in ocular version was approximately half the change in body orientation resulting from the swim (gradient = − 0.43, *R*^2^ = 0.39; Figure 3B, right). By computing the intersection of sight-lines emanating from the eye before and after the saccade-swim event, we estimated that these opposing conjugate saccades serve to stabilize a visual plane ~17 mm (~4 body lengths) from the animal (Figures 3G and S3E). Thus, zebrafish appear to use small conjugate saccades to compensate for body rotation and thereby maintain stable visual perception of nearby objects during forward locomotion.

In summary, larval zebrafish use conjugate saccades in four contexts (Figure 3H): (1) fast phases of the optokinetic nystagmus serve to recenter eye position; (2) large-amplitude saccades are generated by stationary animals, likely subserving visual exploration; (3) saccades coincident with body turns of the same laterality are used to shift gaze; and (4) small saccades in the opposite direction to body rotation help to maintain gaze during forward locomotion.

### Convergent saccades enable precise gaze shifts to foveate prey

A defining feature of zebrafish hunting behavior is that all hunting routines commence with a convergent saccade and a high-vergence angle is then sustained throughout prey tracking.^17,18^ It has been suggested that increasing the extent and proximity of the binocular visual field may support a simple stereopsis mechanism for judging distance to prey at the moment immediately prior to capture strikes.^17^ However, when larvae first initiate hunting, convergent saccades are often lateralized toward prey,^18,29^ raising the possibility that they help to binocularly visualize the target from the earliest stages of prey pursuit. We therefore examined how saccades are coordinated with body movements during orientations to prey.

**Figure 4:**
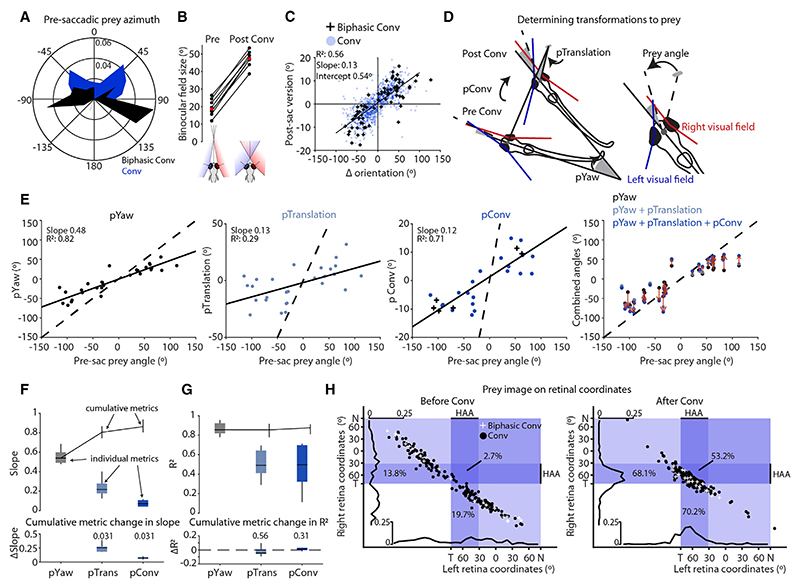
Convergent saccades foveate prey from the onset of hunting (A) Prey azimuth at the time of the convergent saccade that defines the onset of a hunting epoch (202 Conv and 25 BConv from eight fish). (B) Median binocular field size pre- and post- saccades for *n* = 8 fish. Median across animals in red. (C) Post-saccadic version versus change in body orientation, with least squares regression fit (*n* = 1,178 saccades from eight fish). (D) Schematic illustrating how pYaw, pTrans, and pConv are computed for the orienting response to prey. Angles corresponding to each metric shown by gray shaded regions. Right: schematic illustrating gaze-referenced prey position, defined as the angle between the vectors connecting the midpoint between the eyes to (1) the prey target and (2) the nearest point of binocular overlap. (E) pYaw, pTrans, and pConv versus pre-saccadic prey position for the first orienting responses of 28 hunting epochs from one example fish. Linear fits, with slope and *R*^2^, shown as solid lines. Right-most panel shows combined eye-body orienting response (by summing pYaw, pTrans, and finally pConv); red arrows indicate change from pYaw to full response. In all panels *y* = *x* shown as dashed line. (F and G) Upper: slope (F) and goodness-of-fit (G) of regression fits to individual (boxplots) and cumulative (shown as median and IQR) prey orientation metrics versus pre-saccadic prey angle. Lower: change in slope (F) and goodness-of-fit (G) with addition of pTrans followed by pConv. *p* values, signed-rank test. *n* = 6 fish. (H) Prey image in naso-temporal retinal coordinates before (left) and after (right) the first orienting response (188 hunting sequences from 6 fish). Each eye is assumed to have a field of view of 163°.^30^ and a high acuity area spanning 50° of temporal retina.^31^ Percentage of prey targets seen by HAA of one or both eyes are shown. See also Figure S4.

Zebrafish initiated hunting routines using both regular and biphasic convergent saccades (Figure 4A). Regular convergent saccades were used when prey was located in the anterior visual field (mean azimuth = 0.4°, mean absolute azimuth = 52.9°), whereas responses to more eccentric prey involved left- or right-directed biphasic convergent saccades (mean azimuth = 1.8°, mean absolute azimuth = 88.2°). By increasing vergence, convergent saccades more than doubled the size of the binocular visual field (from 19° to 47°) (Figure 4B). In addition, post-saccadic version smoothly covaried with body reorientation, such that larger turns were associated with more lateralized egocentric gaze (Figure 4C). Thus, convergent saccades have both vergence and version components that increase the extent of the binocular visual field and horizontally shift gaze in cooperation with body movements, respectively.

To assess how larvae control convergent saccades and accompanying body movements in the context of goal-directed predatory gaze shifts, we decomposed the first orienting response toward prey (188 individual hunting epochs from six animals). We mapped prey position (angle in the horizontal plane) to a gaze-referenced coordinate system (Figure 4D, inset) and evaluated the specific contributions of body rotation, body translation, and the saccadic eye movement to redirecting gaze toward the target (Figure 4D). We found that zebrafish smoothly modulated all three components in accordance with prey position (Figures 4E and 4F). Body rotation (pYaw) made the largest contribution to the change in gaze-referenced prey position, with a magnitude of approximately half of initial prey azimuth (gain = 0.54 [0.49–0.60], median [IQR], *N* = 6 fish). Translation of the head resulting from the first swim bout (pTrans) further served to align the frontal axis with the prey target, with gain of 0.22 (0.18–0.28). Finally, the saccadic eye movement provided an additional goal-directed gaze shift (pConv, gain = 0.07 [0.04−0.11]). As a result, the combined eye-body movement shifted binocular gaze toward the target prey with an overall gain of 0.86 (0.80−0.94).

To estimate how these three components impact the precision of the orienting response, we assessed the goodness-of-fit (*R*^2^) of linear fits to prey position, while successively including the pYaw, pTrans, and pConv components of individual reorientations (Figure 4G). As well as having the highest gain, pYaw was the most precise motor component, having a median *R*^2^ of 0.86 (0.82–0.92). pConv and pTrans were less accurate but did not significantly affect the overall precision of the goal-directed visual orientation (Figure 4G).

Next, we tested the hypothesis that the high gain of the orienting response is sufficient to binocularly foveate prey from the onset of hunting. Zebrafish larvae have a fovea-like high acuity area (HAA) in the ventral-temporal retina with an elevated density of UV cones that is thought to be crucial for visualizing UV-scattering prey.^31,32^ By projecting prey location into retinal coordinates (Figure S4A), we estimated that for the vast majority of hunting epochs, the first orientating movement shifted the image of prey to the HAA of at least one eye (86.0% [78.6%−88.9%], *n* = 6 fish); moreover, in half the epochs, this first maneuver was sufficient to binocularly foveate prey (54.4% [41.4%−57.1%]) (Figure 4H).

In sum, convergent saccades are coordinated with body movements to foveate prey from the onset of hunting.

### Distinct timing rules for binocular and eye-body coordination across saccade types

**Figure 5:**
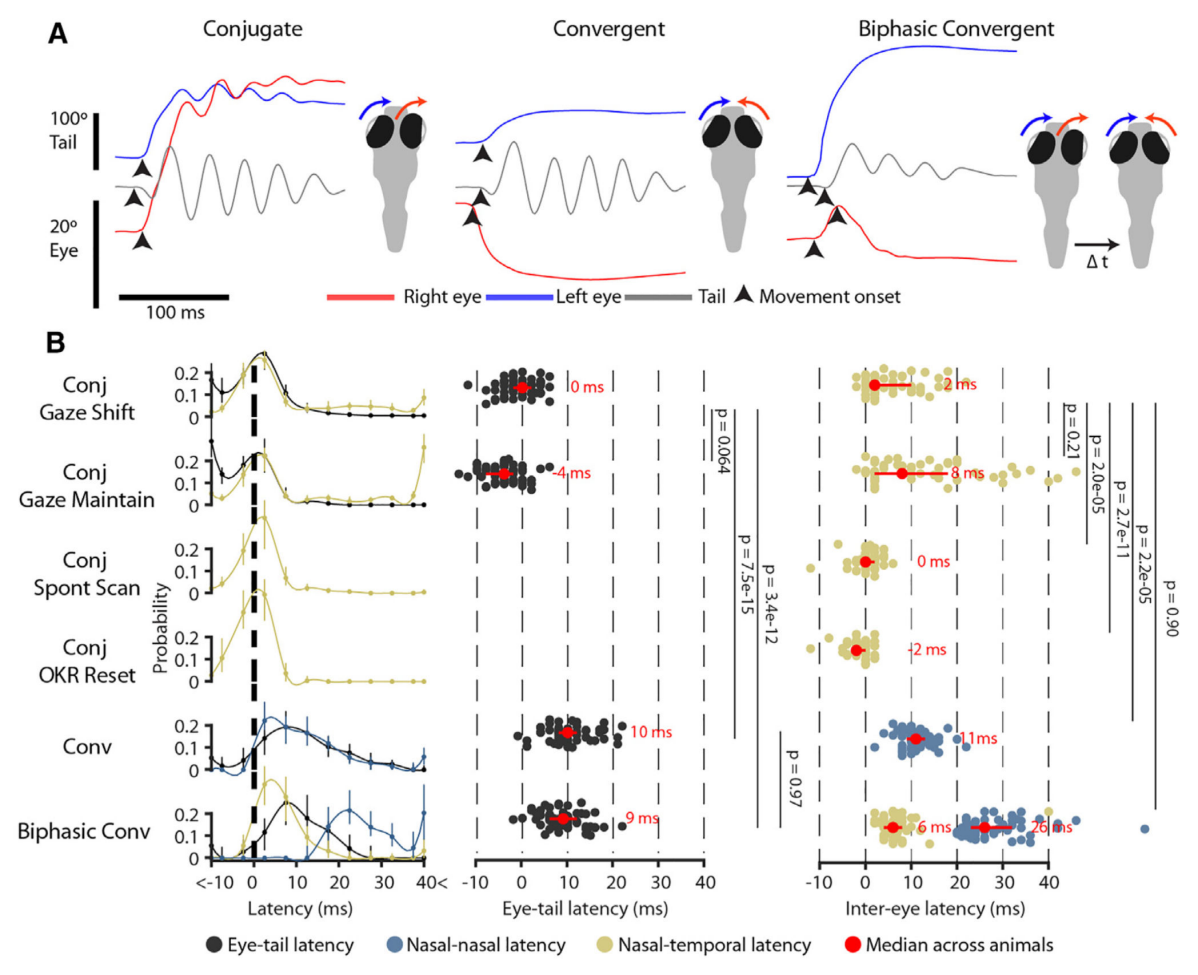
Timing relationships between eye and tail movements vary across saccade types (A) Movement onset times (arrowheads) for the eyes and tail for exemplar conjugate, convergent, and biphasic convergent saccades. (B) Left: distribution of eye-tail and inter-ocular latencies across saccade types. All latencies measured relative to first nasal eye rotation. Data shown as median (IQR) proportions across *n* = 58 animals, with spline fits. Middle, right: median eye-tail (middle) and inter-ocular (right) latencies per animal. Red points and bars show grand median (IQR) across animals. *p* values from Kruskal-Wallis with Dunn-Sidak post-hoc tests. See also Figure S4.

When coordinated eye and body movements redirect gaze, saccades typically precede head/body rotations by a few milliseconds. However, relative timing depends on several factors, including task requirements.^33^ Because conjugate and convergent saccades were coordinated with swims in distinct contexts, we examined movement timing to gain insight into whether there might be differences in the organization and coordination of the underlying neural commands. For this analysis, we used high-temporal-resolution (300 Hz) tracking data from tethered larvae to estimate inter-ocular and eye-tail latencies (Figures 5A and S4B; STAR Methods).

The precise timing of eye and tail movements differed across saccade types (Figure 5B). For conjugate saccades occurring in the absence of tail movements (scanning saccades in stationary animals and OKR fast phases), the latency between movements of the two eyes was small (≤5 ms for 69.6% [60.0% −78.1%] saccades; median [IQR] across *n* = 58 animals). However, when conjugate saccades were accompanied by swims, inter-ocular latencies were substantially longer, and nasal eye movement preceded temporal eye movement (inter-ocular latency 4 [2–10] ms; *p* = 6.2 × 10^−10^, signed rank test versus zero). Tail movement was approximately coincident with the first eye rotation (eye-tail latency −2 [−4 to 0] ms; *p* = 6.6 × 10^−4^). We did not observe significant differences in these timing relationships for saccades that maintain gaze during forward locomotion versus those that shift gaze in coordination with turns (Figure 5B).

Biphasic convergent saccades comprise an initial conjugate rotation followed by the abducting eye reversing direction and moving nasally (right eye in example in Figure 5A). We found that the initial conjugate movement had inter-ocular latency of 6 (4–8) ms, which was not significantly different to conjugate gaze-shifting saccades (Figure 5B). The second nasal rotation then followed with a long delay (26 [23–32] ms). By contrast, regular convergent saccades showed significantly longer interocular and eye-tail latency compared with conjugate saccades (inter-ocular, 11 [9–13] ms; eye-tail, 10 [8–12] ms).

In sum, saccadic eye movements of larval zebrafish are coordinated with body movements on a millisecond timescale. As in other species, latencies are incompatible with sensory feedback and instead indicate that eye and body movements are controlled by a common neural command. However, timing relationships differ across saccade types, implying that distinct patterns of circuit activity coordinate the two eyes and, in particular, the eyes and body for convergent versus conjugate saccades.

### Velocity profiles and main sequence relationships indicate distinct extraocular motoneuron activity controls different saccade types

To produce a saccade, extraocular motoneurons generate a stereotypical pulse-glide-step firing profile in which a burst of high-frequency spiking (pulse) first accelerates the eye and firing rate then decays (glide) to a lower and sustained rate (step) that holds the eye in its new position against centripetal elastic forces.^3^ Due to the regular properties of the ocular plant, features of motoneuron activity can be readily inferred from the kinematics of the eye movement.^4,34,35^ We therefore used high-temporal-resolution tracking data to assess the kinematics of conjugate and convergent saccades to estimate if they might be produced by distinct brainstem motor control signals (*n* = 58 tethered larvae).

**Figure 6:**
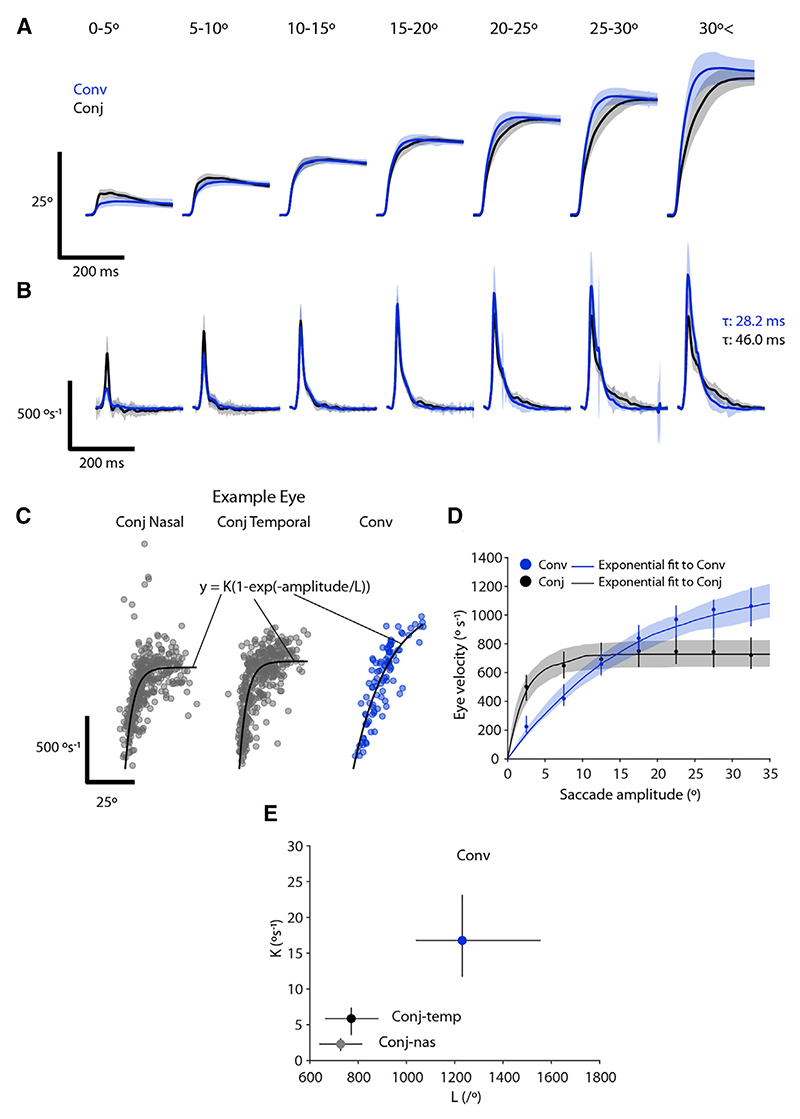
Distinct velocity main sequence relationships for convergent and conjugate saccades (A and B) Eye position (A) and velocity (B) time series for adducting (nasal) saccadic eye movements from convergent and conjugate saccades of different amplitudes. Mean ± SD for *n* = 116 eyes from 58 fish. (C) Velocity main sequence relationship for one example eye with exponential fits. (D) Average velocity main sequence for convergent and conjugate adducting saccades. Lines and shading show median (IQR) across exponential fits to *n* = 116 eyes from 58 fish. For reference, points and lines indicate median (IQR) eye velocity per amplitude bin. (E) Fit coefficients for saccade types (median with IQR). See also Figure S5.

Conjugate and convergent saccades had distinct eye position and velocity time courses. When comparing the adducting saccadic eye movements that are common to both, we observed that for amplitudes >15°, the eye reached its final position more quickly for convergent saccades, whereas conjugate saccades took longer to obtain final eye position (Figure 6A). Convergent adducting saccades had rather symmetrical velocity profiles and peak velocity progressively increased with amplitude (Figure 6B). By contrast, peak velocity was substantially lower for large conjugate saccades, and velocity profiles were markedly asymmetric, with a protracted tail. These features indicate that conjugate saccades are hypometric, showing a dynamic undershoot and then slowly obtaining final position. We considered whether these differences might be explained by systematic differences in initial eye position in the orbit; however, when we binned saccades by starting position, the same hypometric pattern for conjugate saccades was observed (Figure S5A). From these observations we can infer that for large conjugate adducting saccades, the “pulse” on medial rectus motoneurons is poorly matched to the required change in eye position.

A defining feature of saccadic eye movements is the “main sequence” relationship, wherein eye velocity increases as a function of saccade amplitude before saturating.^36^ To examine this, we fit exponential functions^37,38^ to the velocity and amplitude of saccades of individual eyes (Figure 6C) and then computed average main sequence relationships (Figure 6D). This revealed that the adducting eye follows distinct main sequence relationships during conjugate versus convergent saccades (Figure 6E; Aikake information criterion [AIC] = 98.1% [97.3%–98.8%], median [IQR] across *n* = 83 eyes, *p* = × 10^−14^, signed-rank test). Specifically, although small conjugate saccades are faster than similarly sized convergent saccades, conjugate saccade velocity saturates rapidly, reaching a plateau of ~700°/s above ~10°. Notably, both gaze-maintaining and gaze-shifting conjugate saccades followed the same main sequence (Figure S5B), supporting the idea that the former are true saccadic eye movements and that all conjugate saccades are generated by a common underlying pattern of neural circuit activity. By contrast, for convergent saccades, velocity increased as a more linear function of amplitude (Figure 6D). These results reveal different patterns of medial rectus motoneuron population activity for the two saccade types. Specifically, the pulse component saturates for conjugate saccades, such that peak velocity fails to keep pace with amplitude but continues to increase for convergent saccades.

We also analyzed biphasic convergent saccades to assess if all three component eye movements were true saccades and, if so, whether they were generated by neural control signals similar to convergent or conjugate saccades (Figures S5C–S5E). We observed that the first nasal eye movement followed the same main sequence relationship as regular convergent saccades (Figure S5D, left). By contrast, the temporal movement of the opposite eye (amplitude 4.0 [3.0–5.4]°, median [IQR]) followed a velocity main sequence comparable to conjugate saccades (Figure S5D, right). This was also supported by linear (rather than exponential) fits of saccade velocity versus amplitude, which showed comparable slopes for convergent saccades and the first nasal component of biphasic saccades (slope ~45/s), whereas the first temporal movement had a much greater slope, equivalent to small amplitude conjugate saccades (~90/s) (Figure S5E). The second nasal movement followed a main sequence with lower velocity compared with regular convergent saccades, likely a result of the immediately preceding temporal rotation. Altogether, this analysis indicates that biphasic convergent saccades comprise three saccadic eye movement components that are likely controlled by monocular premotor commands (see discussion).

## Discussion

By analyzing thousands of rapid eye movements in tethered and freely swimming zebrafish larvae, we identified five saccade types that differ in oculomotor kinematics and binocular coordination and which are deployed in a context-dependent manner. We defined four roles for conjugate saccades, including coordination with swims of either the same or opposite laterality to shift or maintain gaze, respectively, during locomotion. By contrast, convergent saccades play a specialized role in generating precise gaze shifts that enable zebrafish to foveate their prey from the onset of hunting. Conjugate and convergent saccades differed in the precise timing of binocular and eye-body coordination and followed different velocity main sequence relationships, pointing to differences in underlying physiological control. Our work aligns with recent efforts to characterize active vision during naturalistic behavior^5,39^ and complements recent studies in zebrafish that have comprehensively defined the animal’s locomotor repertoire^26^ and determined how swims are selected and sequenced during exploration and hunting.^14,40–43^ By uncovering when and how saccades are used to redirect gaze, this study provides insight into the visuomotor strategies that organize behavior and will guide experiments examining circuit control of eye movements, eye-body coordination, and visuomotor processing. Finally, we note that our estimate of the zebrafish saccadic repertoire may be incomplete. We assayed only a subset of (visually guided) behaviors and restricted our analysis to horizontal eye movements. Analysis of a broader range of behaviors and tracking of vertical and torsional eye movements^44^ may reveal additional types of, or uses for, saccades, perhaps in coordination with pitch/roll postural adjustments.^45^

### Conjugate saccades and visual exploration

Zebrafish used conjugate saccades alone to redirect gaze when stationary and to reset eye position (i.e., fast phases) during the optokinetic nystagmus and coordinated saccades with swims to either shift or maintain gaze direction. In our classification, conjugate saccades were characterized by both eyes rotating in the same direction, but the amplitude of left and right eye movements were not necessarily equal. Dissimilar amplitudes were clearly observed for the large conjugate saccades of stationary animals, where the abducting saccade was typically greater. By producing a slight divergence, these saccades expand the visual field, compatible with the idea that they allow the animal to survey a broad region of its environment even when at rest. These scanning saccades will reduce the (time-averaged) extent of the blind spot in the visual field behind the animal^17^ and may be part of an active sensing strategy, for example, to sample luminance gradients.^14^ An alternative, non-mutually exclusively hypothesis, is that these saccades help overcome visual adaptation.^46^ In any case, it is clear that similar to other fish species,^47–49^ stationary zebrafish use saccadic eye movements alone to redirect gaze.

Conjugate saccades are coordinated with head/body movements in both foveate and afoveate species (the “afoveate saccadic system”^2^), and accordingly, we found that larval zebrafish head/body reorientations exceeding ~10° were paired with conjugate saccades of the same laterality. In contrast to primates,^33^ gaze shifts of increasing amplitude were not accomplished by systematically varying the relative contributions of the eyes versus head/body. Instead, body reorientation scaled linearly with the overall gaze shift and the change in ocular version was quite variable. However, as in other species, post-saccadic eye position was displaced in the direction of locomotion. By contrast, forward swims were coincident with conjugate saccades of the opposite laterality. This was surprising, because, to our knowledge, saccades paired with head/body movements of opposite directionality have not been described previously. We believe that these are bona fide saccadic eye movements because they conform to the velocity main sequence and precede the spino-ocular coupling reflex that compensates for head yaw during swimming. By opposing body reorientation, these saccades help to maintain the animal’s line of sight during forward locomotion; therefore, we refer to them as gaze-maintaining. Although an amplitude of approximately half of the body reorientation might at first glance appear insufficient, as noted by Easter et al.,^50^ “perfect” compensation would only serve to stabilize a visual plane at infinity, which is likely of little use in scattering aquatic environments. Instead, we estimated that on average, gaze-maintain saccades stabilize a visual plane approximately 17 mm (~4 body lengths) from the animal. Objects at this distance will appear stationary (whereas those further away will appear to move in the direction of locomotion, and those closer will move in the opposite direction), which may both support stable visual perception and provide motion parallax cues during navigation. Calcium imaging experiments have identified activity in rhombomeres 2 and 3 correlated with the direction of tail and eye movement,^13,14,40^ but because eye and body rotations were highly correlated in these assays, it is unclear if this brain region controls either or both motor outputs. The switch in saccade directional contingency described here creates a clear distinction between forward swims and turns and provides a handle for future studies to dissect the neural commands that generate coordinated eyebody motor programs.

### Convergent saccades, binocular vision, and foveation of prey

Convergent saccades are a defining feature of larval zebrafish hunting behavior.^17,18,24^ Both regular and biphasic convergent saccades obtained eye velocities in excess of 600°/s; these eye movements are therefore unlike the slow (~30°/s) fusional vergence movements of primates and instead more similar to primate disjunctive saccades, which are used to rapidly shift fixation between points at different distances and directions in three-dimensional space.^51,52^ Zebrafish use these specialized saccadic eye movements to engage a predatory mode of gaze during hunting and a high-vergence angle is then sustained throughout prey tracking until after the final capture strike. Immediately prior to capture, the eyes are symmetrically and maximally converged such that the most proximal point of binocular overlap is directly ahead of the larva and only 400 μm from the midpoint of the eyes.^17^ On the basis of these observations, we proposed that eye convergence likely supports a simple stereopsis mechanism, allowing larvae to estimate that prey is located at a specific point in egocentric space (the “strike zone”) and release a capture swim, a hypothesis that has received support from elegant lens-removal experiments.^42^ However, because convergent saccades occur at the onset of hunting and are lateralized toward prey,^18,29^ it seems likely that binocular vision plays additional roles throughout prey tracking. Here, we find that zebrafish smoothly control the conjugate (version) component of convergent saccades in accordance with retinotopic prey azimuth. By analyzing prey position in a gaze-referenced coordinate space that is relevant for visual perception, we found that the combined eye-body gaze shift redirects the binocular visual field toward prey with surprisingly high gain (~0.9). The effect of this “visual grasp” is to bring prey images to the HAA (“fovea”) of the retina, which contains an elevated density of UV cones and additional physiological specializations for detection of UV-bright prey.^31^ This suggests that convergent saccades are part of a visuomotor strategy to achieve high signal-to-noise detection of low-contrast prey objects. Moreover, in half the hunting sequences, the first orienting maneuver was sufficient to bring prey images to the HAA of both eyes. Beyond further increasing detection sensitivity (by a factor of up to $\sqrt{2}$), it seems plausible that binocular foveation, along with an internal (efference copy) estimate of eye position, allows larvae to estimate prey distance by a simple algorithm equivalent to triangulation. In support of this, swim vigor is modulated by prey distance during prey tracking (at distances <4 mm),^41^ indicating that larvae have the means to estimate this variable. Whereas conjugate saccades were near-coincident with tail movements, convergent saccades led the tail by 10 ms. It has been suggested that moving the eyes first helps to compensate for the sluggishness of visual processing during gaze shifts^2^; such a function would therefore imply a particular importance for maintaining prey perception during hunting.

### Saccade-type-specific neural control

A key finding of our study is that conjugate and convergent saccades obeyed distinct velocity main sequence relationships and displayed differences in the millisecond coordination of the eyes and tail, together suggesting differences in underlying neural control. Although these observations were made in tethered larvae (a necessity to obtain high-quality tracking data) and we cannot rule out the possibility that specific kinematics and/or coordination patterns might differ in unrestrained animals, it nonetheless seems likely that they reveal the existence of saccade-type-specific circuitry. Modulation of saccade latency and kinematics has been previously shown in several contexts,^33,53–55^ and the slope of the velocity main sequence increases when saccades are accompanied by a coordinated arm reach^56^ and decreases with extended time on task or following sleep deprivation.^57^ A velocity main sequence has previously been described for spontaneous conjugate saccades and OKR fast phases in larval zebrafish,^16,58^ and our results extend these findings to show that all subtypes of conjugate saccade (including gaze-shift and gaze-maintain saccades paired with swims) conform to the same main sequence relationship. This, in turn, suggests that all conjugate saccades are controlled by the same peripheral circuits, in line with established ideas about saccade generation.^36,55,59^ This velocity main sequence showed an inflection point at ~10°, similar to saccades in other species, including goldfish^60^ and humans.^38^ At greater amplitudes, there is minimal further increase in peak eye velocity, which indicates that the phasic pulse component of extraocular motoneuron activity reaches a ceiling and is no longer able to scale with the amplitude of the gaze shift. This is concordant with the pronounced asymmetry we observed in the velocity time course, where after an initial rapid acceleration, hypometric conjugate saccades obtained final eye position with a much slower, “glissadic” eye movement, likely during the slide and/or step phase of motoneuron firing. Here, it is pertinent to note recent work that has revealed a very broad range of time constants in extraocular motoneuron activity matched to a similarly broad range of viscoelastic time constants in the oculomotor plant.^61^ In future, it would be interesting to apply these models to the various types of saccadic eye movement we describe to better estimate underlying neural activity. Strikingly, convergent adducting saccades had markedly different velocity profiles and main sequence relationships. Although small (<10°) saccades were slower than equivalent conjugate saccades, peak velocity continuing to increase across the full dynamic range of amplitude (~35°), indicating that the pulse on medial rectus motoneurons was matched to the required change in eye position.

What might be the physiological basis for these kinematic differences? One possibility is that there is a distinct (or additional) population of medial rectus motoneurons responsible for nasal eye rotations during convergent saccades. However, although it has been long debated, there is currently little evidence for motoneurons with specialized roles in particular types of eye movement, let alone specific subtypes of saccade. Although there are two major types of extraocular motoneurons, with distinct molecular properties, patterns of afferent input, and synapse termination on extraocular muscle fibers, physiological data reveal a smooth continuum of functional properties, and it is generally assumed that all extraocular motoneurons participate in all classes of eye movement.^62–64^ Nonetheless, extraocular motoneurons do show substantial variation in recruitment threshold and position and velocity sensitivity. Therefore, differences in afferent input may give rise to distinct patterns of recruitment to generate saccadic eye movements with type-specific kinematics. In future, neural activity recordings in the zebrafish brainstem will be a powerful tool to discover saccade-type-specific neural populations and evaluate motoneuron activity as a function of saccade type and kinematics.

### Biphasic convergent saccades

Finally, the unusual properties of biphasic convergent saccades warrant special mention. These comprise three closely coordinated saccadic movements: an initial conjugate eye rotation, shortly followed by reversal of the abducting eye. Closely spaced saccades have been described in humans,^65^ and the reversal in biphasic convergent saccades is perhaps most reminiscent of dynamic overshoot, a common phenomenon in which a primary saccade is immediately followed by a small secondary saccade in the opposite direction.^66,67^ Dynamic overshoot is also a monocular phenomenon, typically of the abducting eye, and the return eye movement has saccadic velocity. It has been suggested that a braking pulse of neural activity normally functions to bring the eye to rest at the end of a saccade and dynamic overshoot may occur when this pulse is excessively large.^67^ Along similar lines, biphasic convergent saccades might arise due to errors in the amplitude and/or timing of multiple saccadic commands that normally control (routine) convergent saccades. The nature of the premotor commands that control binocular eye movements has long been debated^68,69^: Hering’s Law posits that both eyes receive identical (conjugate and vergence) neural commands, whereas Helmholtz argued for independent control of each eye. The fact that the first nasal and temporal eye movements of biphasic convergent saccades conform to different main sequence relationships (characteristic of convergent and conjugate saccades, respectively) seems compatible with emerging evidence in favor of a monocular control framework.^70–73^

## Resource Availability

### Lead Contact

Further information and requests for resources and reagents should be directed to and will be fulfilled by the lead contact, Isaac H. Bianco (i.bianco@ ucl.ac.uk).

### Materials availability

Constructs and transgenic lines generated in this study are available from the lead contact on request.

## Star★Methods

Detailed methods are provided in the online version of this paper and include the following:

- KEY RESOURCES TABLE
- EXPERIMENTAL MODEL AND STUDY PARTICIPANT DETAILS
  ○ Zebrafish lines and care
- METHOD DETAILS
  ○ Behavioral tracking in tethered larvae
  ○ Behavioral tracking in freely swimming larvae
  ○ Saccade detection and classification
  ○ Swim kinematic analysis
  ○ Contextual deployment of saccades
  ○ Orientating responses to prey in freely swimming larvae
  ○ Latency and velocity main sequence analyses
  ○ DNA cloning and transgenesis
- QUANTIFICATION AND STATISTICAL ANALYSIS

## Star✶Methods

### Key Resources Table

**Table:** 

REAGENT or RESOURCE	SOURCE	IDENTIFIER
Deposited data		
Tethered and free-swim saccade data	This paper	Mendeley Data, V1: https://doi.org/10.17632/vd5zdfwc37.1
Experimental models: Organisms/strains		
Tg(elavl3:H2B-GCaMP6s)jf5Tg	Freeman et al.^74^	ZFIN: ZDB-ALT-141023-2
Tg(pvalb6:KalTA4)u508Tg	Antinucci et al.^75^	ZFIN: ZDB-ALT-200519-9
Tg(UAS:GCaMP6f)icm06Tg	Bohm et al.^76^	ZFIN: ZDB-ALT-160119-5
Tg(isl1:GFP)rw0Tg	Higashijima et al.^77^	ZFIN: ZDB-ALT-030919-2
Tg(vsx2:Gal4FF)nns18Tg	Kimura et al.^78^	ZFIN: ZDB-ALT-130617-1
Tg(elavl3:GCaMP7f)u343Tg	This paper	N/A
Tg(UAS:RFP)idv3Tg	Auer et al.^79^	ZFIN: ZDB-ALT-230307-8
Software and algorithms		
MATLAB 2021a	MathWorks	https://uk.mathworks.com/products/new_products/release2021a.html
UMAP	Meehan et al.^80^	https://uk.mathworks.com/matlabcentral/fileexchange/71902-uniform-manifold-approximation-and-projection-umap
LabView	National Instruments	https://www.ni.com/en/shop/labview.html
Psychophysics Toolbox	Brainard^81^	http://psychtoolbox.org/
DBSCAN	Script written by authors to implement Ester et al.^82^	Mendeley Data, V1: https://doi.org/10.17632/vd5zdfwc37.1
Custom lowess function	This paper	Mendeley Data, V1: https://doi.org/10.17632/vd5zdfwc37.1
Code to plot figures	This paper	Mendeley Data, V1: https://doi.org/10.17632/vd5zdfwc37.1

### Experimental Model And Study Participant Details

#### Zebrafish lines and care

Zebrafish lines were maintained in the Tübingen background. Larvae were reared in fish-facility water on a 14/10 h light/dark cycle at 28.5°C and were fed *Paramecia* from 4 dpf onwards. All larvae carried the *mitfa*^83^ skin-pigmentation mutation. The 152 animals used for tethered behavioral analysis carried transgenes as follows: 76 animals carried Tg(elavl3:H2B-GCaMP6s)jf5Tg.^74^ 60 animals carried Tg(pvalb6:KalTA4)u508^75^ and Tg(UAS:GCaMP6f)icm06.^76^ 4 animals carried Tg(isl1:GFP)rw0Tg^77^ and Tg(elavl3:H2B-GCaMP6s) jf5Tg. 6 animals carried Tg(vsx2:Gal4FF)nns18Tg,^78^ Tg(UAS:RFP)idv3Tg^79^ and Tg(elavl3:H2B-GCaMP6s)jf5Tg. 6 animals carried Tg(elavl3:GCaMP7f)u343Tg. Eight animals were used for free-swimming assays analysis and were not transgenic. The sex of the larvae is not defined at the early stages of development used for these studies. Experimental procedures were approved by the UK Home Office under the Animals (Scientific Procedures) Act 1986.

### Method Details

#### Behavioral tracking in tethered larvae

Larvae were tethered in 3% low-melting point agarose gel in a 35 mm petri dish lid and sections of gel were carefully removed using an opthalmic scalpel to allow free movement of the eyes and tail below the swim bladder. Larvae were allowed to recover overnight before testing at 6 or 7 dpf. Behavior was tracked while animals underwent two-photon calcium imaging using a custom microscope as described in Antinucci et al.^75^ Eye movements were tracked at either 60 or 300 Hz using a FL3-U3-13Y3M-C camera (Point Grey) that imaged through the microscope objective. Tail movements were imaged at 420 Hz under 850 nm illumination using a sub-stage GS3-U3-41C6NIR-C camera (Point Grey). Horizontal eye position and tail posture (defined by 13 equidistant x-y coordinates along the anterior-posterior axis) were extracted online using machine vision algorithms.^24^

Two projectors were used to present visual stimuli. The first (Optoma ML750ST) back-projected stimuli onto a curved screen placed in front of the animal at a viewing distance of 35 mm while the second (AAXA P2 Jr) projected images onto a diffusive screen directly beneath the chamber. Wratten filters (Kodak, no. 29) were placed in front of both projectors. Visual stimuli were designed in MATLAB using Psychophysics Toolbox.^81^ Prey-like moving spots comprised 6° or 12° bright or dark spots (Weber contrast +1 or -1 respectively) moving at 30°/s either left → right or right / → left across 152° of frontal visual space. For dark flashes, both projectors were switched to zero pixel value for 3 s. Looming stimuli comprised expanding dark spots (Weber contrast -1) that simulated an object approaching at constant velocity (10°–70°, L/V 490 ms).^84^ Optomotor stimuli comprised drifting sinusoidal gratings (wavelength 10 mm, velocity 10 mm/s, Michelson contrast 1) presented from below and moving in four cardinal directions with respect to the animal. Optokinetic stimuli comprised drifting sinusoidal gratings (wavelength 19°, velocity 0.3 cycles/s, Michelson contrast 0.5) presented in front of the animal and moved left-to-right and right-to-left. For all experiments, stimuli were presented in a pseudo-random sequence with 30 s inter-stimulus interval. Microscope control, stimulus presentation and behavior tracking were implemented using LabVIEW (National Instruments) and MATLAB (MathWorks).

#### Behavioral tracking in freely swimming larvae

Free-swimming behavior was recorded in a similar manner to Henriques et al.^29^ In brief, behavior was recorded in a 35 mm petri dish with 3% low-melting point agarose placed along the walls to limit thigmotaxis. The chamber was placed on a horizontal platform onto which visual stimuli could be presented (Acer C202i projector) via a cold mirror from below. Images were acquired at 300 Hz under 850 nm illumination using a Mikrotron EoSens 4CXP camera equipped with a machine vision lens (Kowa) and a 850 nm band-pass filter.

Visual stimuli were designed in MATLAB using Psychophysics toolbox. Optomotor stimuli comprised sinusoidal gratings (wave-length 8 mm, velocity 8 mm/s, Michelson contrast 1, duration 6 s) that drifted at 90° to the left or right with respect to the fish, with stimulus direction locked to fish orientation and updated in real-time. Stimuli were presented with a minimum interstimulus interval of 120 s and only when the centroid of the larva was within a predefined central region (~11 mm from arena edge). If the fish strayed out of this region, a concentric grating was presented that drifted towards the center of the arena to attract the fish back. Only behavior data from within this central region was analyzed to avoid tracking errors caused by reflections from the chamber edge.

At the beginning of the experiment 10-30 *Paramecia* were added to the dish to promote hunting behavior.

Eye and tail kinematics were tracked online as described in Henriques et al.^29^ Throughout the experiment a cropped (23.9 mm × 23.9 mm, 13.0 mm/px) movie centered on the centroid of the larva was recorded to allow subsequent analysis of hunting orientations (see below). Each experiment lasted around 45 min. Camera control, online tracking and stimulus presentation were implemented using LabVIEW (National Instruments) and MATLAB (Mathworks).

#### Saccade detection and classification

Raw eye position traces were interpolated onto a 100 Hz time-base and low-pass filtered with a cut-off frequency of 1 Hz. Rapid eye movement events were detected as peaks in the convolution of filtered eye position with a step function (width 160 ms, example trace in Figure 1B), with the timepoint of the peak providing a first coarse estimate of movement initiation time. Rapid eye movement events of the left and right eye that occurred within 100 ms of one another were paired and treated as a single binocular event. After this pairing step, events that occurred within 300 ms of a preceding event were discarded, to limit overlap between windows for calculating saccade metrics (see below) and because manual inspection revealed that these movements were rarely saccadic.

To reliably estimate the eye position and velocity metrics for saccade classification, raw eye position traces were interpolated onto a 500 Hz timebase and smoothed with a custom LOWESS function. A more refined estimate of onset time was determined by convolving smoothed eye position with two step functions of width 100 ms and 40 ms, taking the product between both convolutions and thresholding the output within a 400 ms window spanning the initial estimate of saccade time. The custom LOWESS function was designed to reduce noise in eye position traces without flattening changes in eye position during saccades. This involved applying the MATLAB lowess function with two different spans depending on whether a saccade-like change in eye position was detected. A shorter span was used during stepwise changes in eye position. For free swim data this span was 80 ms. For tethered data no smoothing was done. A larger span was used outside of stepwise changes. This was 133 ms for free swim and 33 ms for tethered data. Stepwise changes were defined by convolving raw eye position traces with a step function and thresholding, in a similar manner to the rapid eye movement detection procedure.

For each rapid eye movement event we evaluated: (a) pre-saccadic eye position, as median eye position during a 200 ms window immediately prior to onset time; (b) max post-saccadic eye position, as the eye position within a 200 ms window starting at onset time that had the greatest absolute deviation from eye position at onset time; (c) median post-saccadic eye position, as median eye position over a 200 ms window starting at the timepoint corresponding to max post-saccadic eye position; (d) eye velocity (cw and ccw), as the maxima and minima, respectively, of the time derivative of eye position, determined by the MATLAB gradient function over a 150 ms window centered at onset time. We then used these measures to calculate nine oculomotor metrics describing each (binocular) rapid eye movement event: *Amplitude* (left and right eye), was the difference between median post-saccadic eye position and pre-saccadic eye position. *Max-median amplitude* (left and right eye), was the difference between max post-saccadic eye position and median post-saccadic eye position and quantifies the degree to which eye position is maintained following a saccade. *Velocity* (cw and ccw for both left and right eye), as described above. *Vergence* was the difference between median post-saccadic eye position of the left and right eye.

To examine variation in oculomotor kinematics and categorize rapid eye movements, we embedded eye movement events in a low dimensional space and applied a density based clustering procedure. To do this, data from each animal was first winsorized (0.5–99.5th percentile) and z-scored. Our initial embedding and clustering was performed using data from tethered larvae, excluding events that initiated during a swimming bout (thus retaining 213,462 of 335,442 events (63.6%) from *N* = 152 fish). This helped to ensure we used high-quality tracking data without artefacts caused by swim-induced changes in eye/head position. Datapoints were embedded into two dimensions using a MATLAB implementation^80^ of UMAP^85^ (run_umap, metric=Euclidean, min_dist=0.11, n_neighbours=199) and the output was clustered using DBSCAN,^82^ with epsilon = 0.34 and minimum point threshold = 570. The number of clusters (8) was manually specified to produce stable and homogenous clusters. Un-clustered points within 3 units of UMAP space to a cluster edge were assigned to a cluster within this radius; the event was assigned to the cluster that had the most successive increases in point density binned along a straight line connecting the event and the cluster centroid. Tethered events that initiated during a swim bout (121,980 events) as well as data from free-swimming larvae (10,569 from 8 fish) were embedded into the same latent space using the UMAP transform method and the same UMAP model. These datapoints were assigned the most common cluster identity from 100 nearest neighbors in the embedding space; however, if those 100 nearest neighbors were separated from the target event by a median Euclidian distance exceeding 0.3 in UMAP space (1,396 tethered events, 0 free-swimming events) then no identity was assigned.

Following this initial clustering we observed that some biphasic convergent saccades were assigned to the Conv cluster, rather than the two BConv clusters. We therefore implemented an additional procedure to detect and reassign BConv events. Specifically, biphasic convergent saccades were defined by having one eye that moved in a temporal direction with velocity and eye displacement exceeding thresholds: The velocity threshold was 60°/s for tethered data and 40°/s for free-swimming data. The eye displacement threshold was one standard deviation of eye position over a 150 ms window terminating 100 ms prior to onset time.

#### Swim kinematic analysis

Raw tail tracking data from tethered larvae comprised 13 x-y centroids defining the midline of the tail. Consecutive centroids define 12 tail segments and vectors of 11 inter-segment angles were computed for each timepoint. Raw tail tracking data from freely swimming larvae comprised 9 x-y centroids, producing 7 inter-segment angles that were interpolated to 11 inter-segment angles to maintain consistency across datasets. Matrices of inter-segment angles over time were interpolated onto a uniform 1000 Hz timebase and smoothed in 2D using a 2-segment-by-7-ms filter. Next, we computed the cumulative sum of inter-segment angles, γ, which was filtered (MATLAB sgolayfilt, order=3, framelength=9) and median subtracted. Thus, changes in tail posture are represented as the time-varying cumulative bend angle along the anterior-posterior axis of the tail: γ_*s*;*t*_, for cumulative inter-segment angle *s* at time-point *t*. To identify swim bouts, we first estimated tail angular velocity, *v*_*t*_ by differentiating γ_11;*t*_, taking its absolute value and filtering (40 ms box-car). We also computed the envelope, *f*_*t*_, as the maximum absolute value of γ_11_;_*t*_ within a 9 ms sliding window. The start of swim bouts were identified at time-points where *v*_*t*_ > 800 deg/s and *f*_*t*_ > 7 deg, and the end of swim bouts was defined when *v*_*t*_ < 200 deg/s and *f*_*t*_ < 10 deg. Bouts less than 61 ms in duration were excluded.

We identified individual halfbeats (leftwards and rightwards excursions of the tail) by finding the maxima and minima of γ_9;*t*_. For tethered larvae we used the sign of the first half-beat for cumulative inter-segment angle 11, *θ*_11;1*st*_, to define swim direction (left/right) and its amplitude as a proxy for swim lateralization. For free-swimming larvae, we computed the change in body orientation (Δ_*ori*_) and displacement (*dx*,*dy*), by taking the difference in body orientation or centroid position 50 ms before and 7.5 ms after a swimming bout; the sign of Δ_*ori*_ defined swim direction.

Rapid eye movements were considered coincident with a swim if their onset time was from 200 ms prior to swim initiation to 200 ms after swim termination.

#### Contextual deployment of saccades

Contexts were defined based upon swim types and visual stimuli and the frequency of saccade types occurring in these contexts was evaluated. The contexts, which are not mutually exclusive, were defined as follows: Swim L/R, swims for which |Δ_*ori*_| ≥ 10° to left/right for free-swimming or |*θ*_11, 1st_| ≥ 25° for tethered fish. Swim F, were swims with |Δ*ori*| (or |*θ*_11,1st_|)below these thresholds. OKR-L/R, optokinetic gratings drifted to the left/right and, unless otherwise stated, there was no accompanying swim bout. Prey spot, presentation of prey-like moving spot stimuli. Orient to spot, first saccade during presentation of prey-like moving spot accompanied by swim with direction corresponding to spot laterality. Loom < 30 deg and Loom > 30 deg, looming stimulus subtended visual angle less than and greater than 30°, respectively. Dark flash, dark flash stimuli. No-stim-no-swim, inter-stimulus intervals and no coincident swim bout.

#### Orientating responses to prey in freely swimming larvae

The initiation of a hunting sequence was defined as a convergent saccade that increased vergence above a threshold. This was determined for each animal by fitting two Gaussians to the bimodal distribution of vergence angles measured across the experiment and setting the threshold to one standard deviation below the center of the higher Gaussian. Next, for each convergent saccade, five consecutive imaging frames (starting 10 ms prior to saccade onset) were assessed for putative prey targets. Putative targets were identified by Gaussian filtering, thresholding and detecting small binary objects (148 < *Area* < 889*μm*^2^ ∩385 μ *Length* < 1540*μm*). If at least one putative target was identified across the five images, target positions were determined manually using a custom MATLAB GUI. Instances where there were multiple prey objects in the animal’s visual field (see below) were not assessed to avoid ambiguity in target identification.

Orientations to prey targets were decomposed into three components: pYaw, was the change in prey angular position attributable to the change in orientation of the larva during the swim bout coincident with the convergent saccade. This was equal to Δ_*ori*_ for said swim bout. pTrans, was the change in prey angular position attributable to translation of the animal’s head during the swim bout coincident with the convergent saccade. This was computed as *α*_*post*_ – *α*_*pre*_, where *α*_*pre*_ was the angle between the vector connecting the midpoint of the eyes and the prey target and the vector defining head orientation at the time of swim initiation. *α*_*post*_ was calculated 7.5 ms after completion of the swim bout as the angle between the vector connecting the midpoint of the eyes and the prey target and the vector defining head orientation *at swim initiation*. In this way, the effect of body orientation change was eliminated. pConv, was the change in the angle of the vector connecting the midpoint of the eyes to the most proximal point of binocular overlap before versus after the convergent saccade. The most proximal point of binocular overlap was defined as the point at which the nasal limits of the left and right visual fields overlapped, with each eye’s visual field taken as 163°.^30^

In Figure 4, linear fits between pYaw, pTrans and pConv components and pre-saccadic prey position were made using the MATLAB fitlm function with robust fit option. Prey position was calculated in gaze-referenced space at convergent saccade onset. To do this, the angle between the vector connecting the midpoint of the eyes and the prey target and the orientation of the head was computed and then the angle of the most proximal point of binocular overlap was subtracted.

#### Latency and velocity main sequence analyses

Saccade initiation time, velocity and amplitude estimates were refined prior to analysis of inter-eye and eye-tail latency (Figure 5) and velocity main sequence (Figure 6). To estimate initiation times, we first computed two eye velocity estimates (Ev and Evsmooth) using raw eye position data up-sampled onto a 500 Hz time-base. Ev was determined by first smoothing eye position with the same custom LOWESS function described above and then computing the time derivative using the MATLAB gradient function. Evsmooth was determined by smoothing eye position with the same custom LOWESS function followed by an additional LOWESS function of span 50 ms, followed by computing the time derivative. The saccade midpoint was defined as the time-point at which Evsmooth peaked within a 160 ms window beginning 20 ms prior to the initial estimate of saccade onset time (see above). Then, saccade initiation was defined as the first time-point where Ev exceeded 100°/s prior to the saccade midpoint.

Saccade amplitude was calculated as the difference between pre- and post-saccadic eye position. Pre-saccadic eye position was the eye position one time-point (2 ms) prior to saccade initiation. Post-saccadic eye position was the median eye position over a 200 ms window starting at the time-point of peak eye displacement post-saccade. Peak displacement was the greatest change in eye position during a 200 ms window starting at saccade initiation, with the direction (positive or negative) determined by saccade type.

Peak eye velocity was maximum/minimum value of Ev during a time envelope that spanned the midpoint of the saccade. The envelope was defined by time-points where Evsmooth exceeded 10°/s.

For biphasic convergent saccades, the reversing eye was analyzed as follows. Initiation of temporal eye movement was defined as the time-point at which Ev exceeded 100°/s in the temporal direction. Initiation of the second nasal rotation was defined as the time-point, following temporal movement initiation, at which Ev was closest to zero. Temporal velocity was the maximum Ev in the temporal direction between temporal and nasal initiation times and the amplitude of the temporal component was difference between eye position at these times. Velocity for the second nasal component was maximum Ev in the nasal direction during a 160 ms window following the initiation time of that movement; amplitude was the difference between post-saccadic eye position (as defined above) and eye position at second nasal movement onset.

Velocity main sequence relationships were fit using the function:

$$velocity\;=K(1-e^{\frac{-ampiltude}{L}})$$

Following Baloh et al.,^37^ where *K* and *L* are constants. Fits were made using the MATLAB fitnlm function. We calculated the Aikake Information Criterion (AIC) to compare models with separate fits to each saccade type versus a single fit to data pooled across types. Fits were made with equal sample sizes from each saccade type (by randomly sampling from the type with more samples) and AIC values were normalized by dividing by the AIC value of the model with one exponential fit to the pooled data.

Linear velocity main sequence fits were made using the MATLAB fitlm function with robust options on and no bias term. For conjugate saccades, we only included saccades of amplitude <10°, to limit the model to the non-saturating portion of the main sequence.

#### DNA cloning and transgenesis

To generate the pTol2-elavl3:jGCaMP7f DNA construct used for creating the Tg(elavl3:GCaMP7f)u343 line, the coding sequence of the genetically encoded calcium indicator jGCaMP7f^86^ (from pGP-CMV-jGCaMP7f, a gift from Douglas Kim and GENIE Project, Addgene 104483) was cloned into the Tol2-elavl3-GCaMP6s backbone^87^ (a gift from Misha Ahrens, Addgene 59531) linearized using AgeI (New England Biolabs) restriction enzyme digestion. The cloning was achieved using the In-Fusion Snap Assembly kit (Clontech/Takara Bio) with the following primers:

elavl3-jGCaMP7-fw: GCAGATAATTACCGGTATGGGTTCTCATCATCATCATCATC elavl3-jGCaMP7-rev: GCCAGGATCCACCGGTTCACTTCGCTGTCATCATTTG

The purified construct (35 ng/μl) was co-injected with Tol2 transposase mRNA (80 ng/μl) into *mitfa* embryos at the one-cell stage. Embryos displaying fluorescence were raised and germline transmission was identified by mating once animals reached sexual maturity. Positive embryos from a single fish were then raised to adulthood and from these a single ‘founder’ fish was selected to establish the Tg(elavl3:GCaMP7f)u343 line.

### Quantification And Statistical Analysis

All statistical analyses were performed in MATLAB. Types of statistical test and *N* are reported in the text or figure legends. All tests were two-tailed and we report *p-value*s without correction for multiple comparisons unless otherwise noted.

## Supplementary Material

Supplemental information can be found online at https://doi.org/10.1016/j.cub.2024.08.008.

Supplemental Information

## Data Availability

The code and pre-processed data to generate the figures in this manuscript are publicly available at Mendeley: https://doi.org/10.17632/vd5zdfwc37.1. The raw data generated in this study will be shared by the lead contact upon request, but they have not been deposited due to their large size.

## Highlights

- Kinematic analysis of thousands of rapid eye movements reveals five saccade types
- Conjugate saccades have at least four identifiable visual functions
- Convergent saccades are coordinated with body movements to foveate prey
- Distinct main sequence relationships imply saccade-type-specific neural control

## In brief

Dowell et al. characterize the repertoire of saccadic eye movements of larval zebrafish and examine their coordination with swims during visuomotor behaviors. Differences in timing, kinematics, and velocity main sequences indicate that different saccade types are controlled by distinct patterns of brainstem activity.
