# Supplementary material for: Kinematically distinct saccades are used in a context-dependent manner by larval zebrafish
Source: Curr Biol. Author manuscript; Available in PMC 2025 Dec 20. (PMC7618497; doi:10.1016/j.cub.2024.08.008)
Supplement: Supplemental Information [file EMS211316-supplement-Supplemental_Information.pdf]

Current Biology, Volume *34*

Supplemental Information

Kinematically distinct saccades are used in a context-dependent manner by larval zebrafish

Charles K. Dowell, Joanna Y.N. Lau, Paride Antinucci, and Isaac H. Bianco

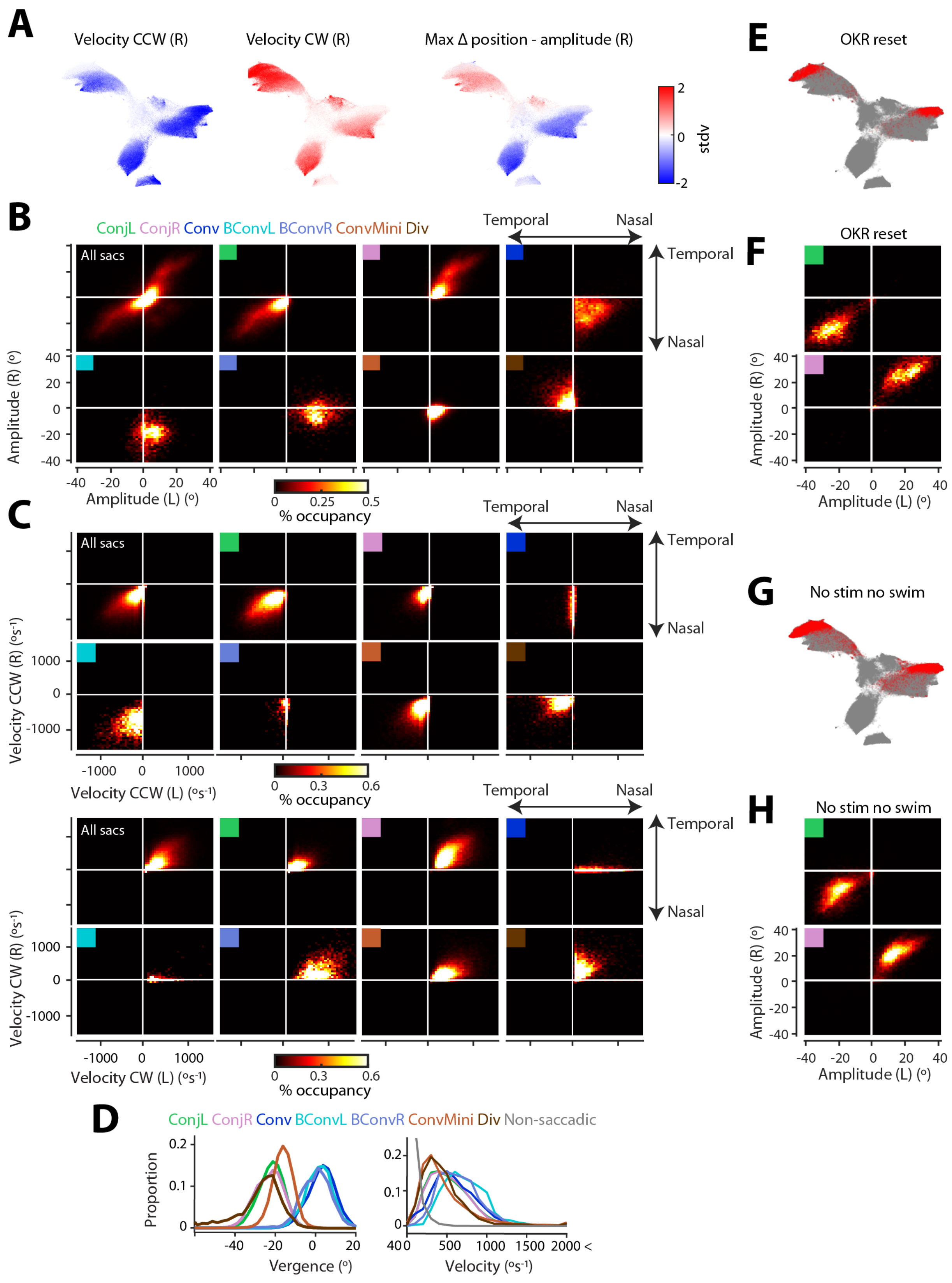

Figure S1: Additional saccade metrics from tethered larvae. Related to Figure 1.

(A) UMAP embedding coloured according to three additional oculomotor metrics. (B–C) 2D histograms of saccade amplitude (B) and velocity (C). Saccade type indicated by coloured key in top left of each panel. (D) Post-saccadic vergence (left) and absolute eye velocity (right), across saccade types. For velocity histogram, non-saccadic cluster is included (grey, see Methods). (E) OKR fast phases (red) overlaid on UMAP space (grey background). (F) 2D histograms of saccade amplitude for OKR fast phases. (G–H) As per E,F for 'spontaneous' conjugate saccades that occurred in absence of stimuli and unaccompanied by tail movement.

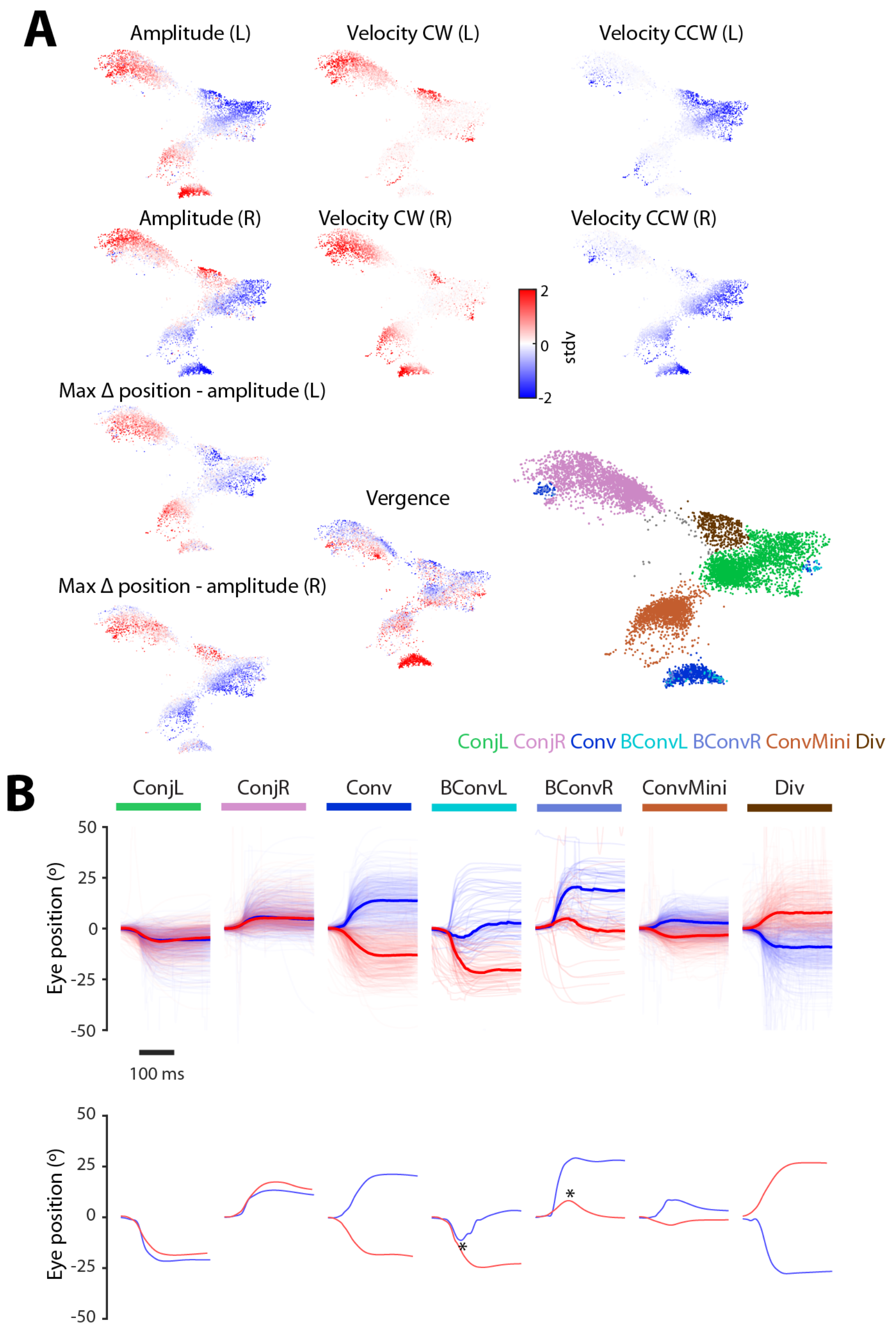

Figure S2: Saccade metrics from freely swimming fish. Related to Figure 1.

(A) Rapid eye movements (9,367 events from 8 fish) after transformation into the 2D UMAP space from Figure 1, coloured by normalised kinematic metrics and saccade type labels. (B) *Top:* For each saccade type, 500 eye position traces are plotted with the median overlaid in bold. *Bottom:* A single example saccade from each type. * indicates reversal of eye velocity during biphasic convergent saccades.

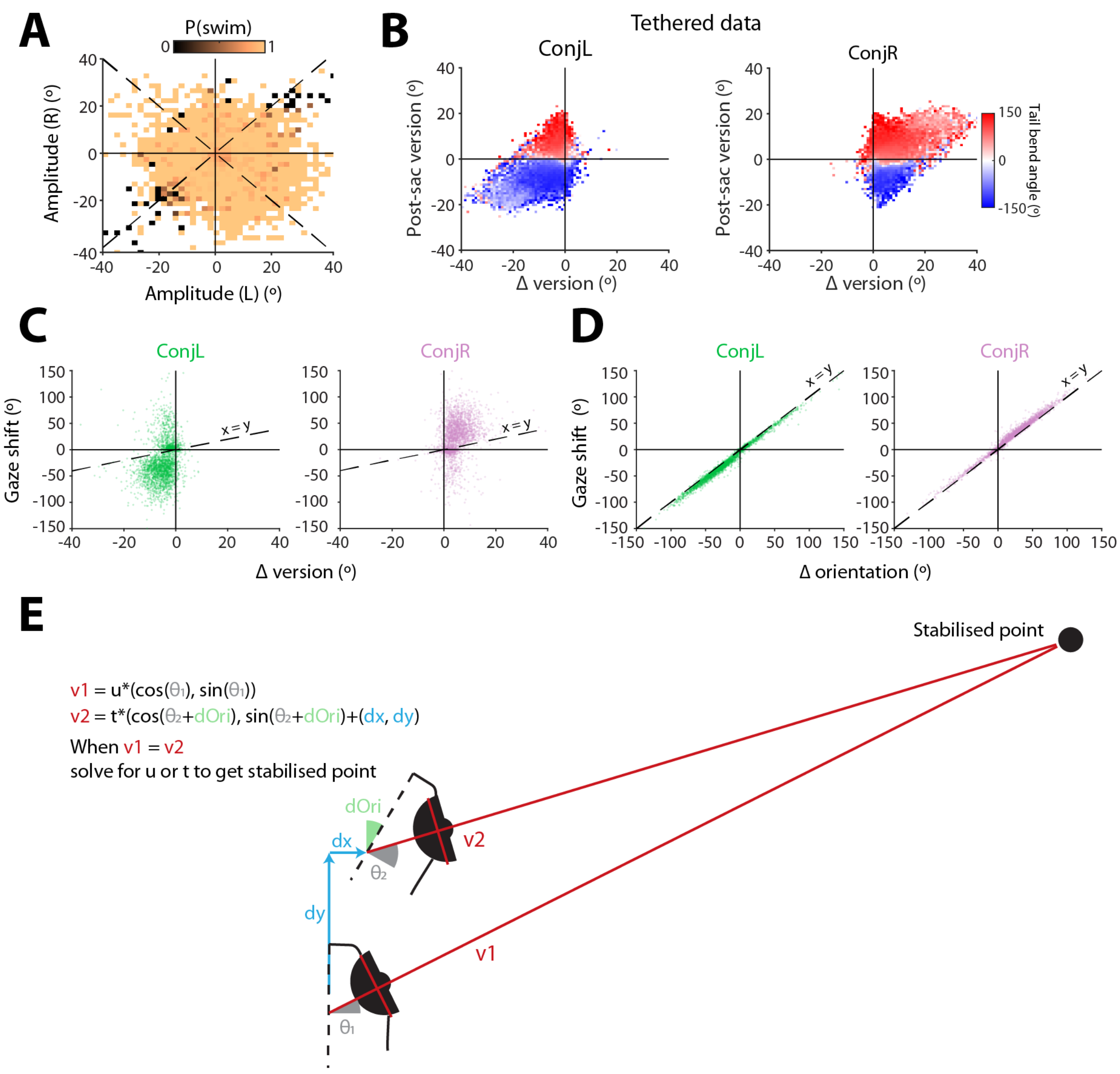

Figure S3: Conjugate saccades – additional data. Related to Figure 3.

(A) Saccade amplitude coded by the probability of a coincident swim (data from 8 freely swimming larvae). (B) Left and right conjugate saccades binned by version change and post-saccadic version and colour-coded by median tail bend angle (152 tethered fish). (C–D) Gaze shift (the sum of version and body orientation changes) versus change in version (C) or change in body orientation (D) (5,869 saccades from 8 freely swimming fish). (E) Calculation of distance of stabilised point for gaze-maintain saccades. Vectors $v1$ and $v2$ describe the eye-in-space sight lines before and after the saccade-swim event; the intersection of these vectors is computed to identify a stabilised point that will be imaged at a constant retinotopic location.

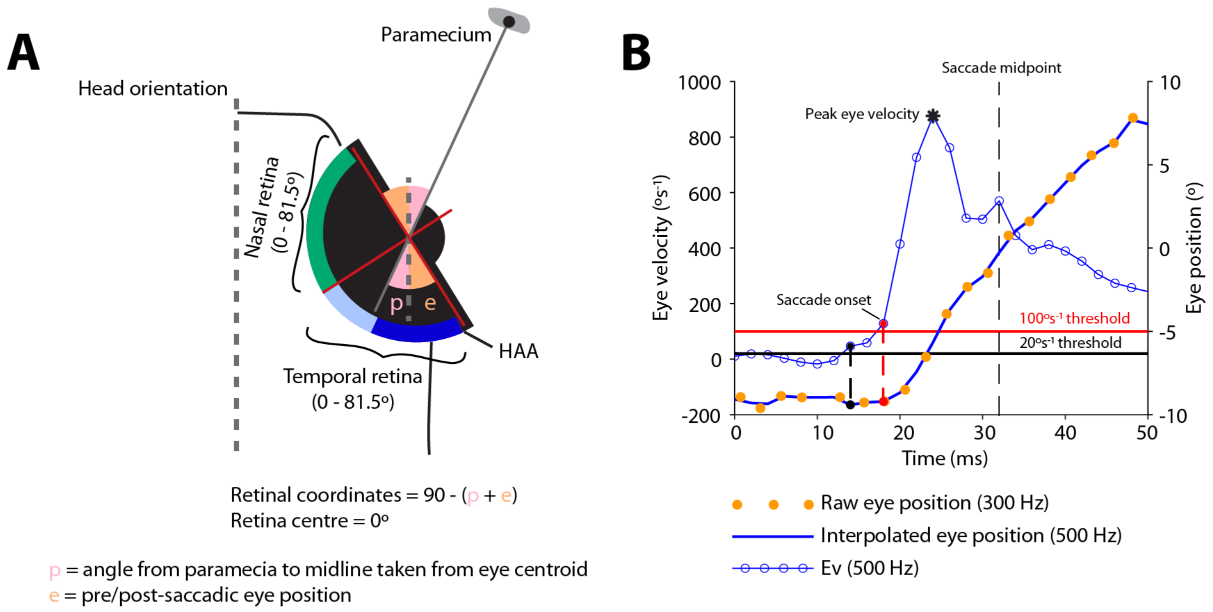

Figure S4: Estimation of retinal images of prey and saccade timing. Related to Figures 4 and 5.

(A) Schematic illustrating projection of prey targets into retinotopic coordinates. e is eye-in-head angular position. p is the angle between a vector connecting the midpoint of the eye to the prey centroid (grey solid) and the head orientation vector (grey dashed). The angular extent of the retina is assumed to be 163 degrees and the HAA 50 degrees. (B) Example saccade illustrating estimation of initiation time. Raw eye position samples, interpolated eye position and estimated eye velocity (Ev) are plotted. Saccade onset is defined as the earliest timepoint at which Ev exceeds a threshold ($100°/s$). For further details see Methods.

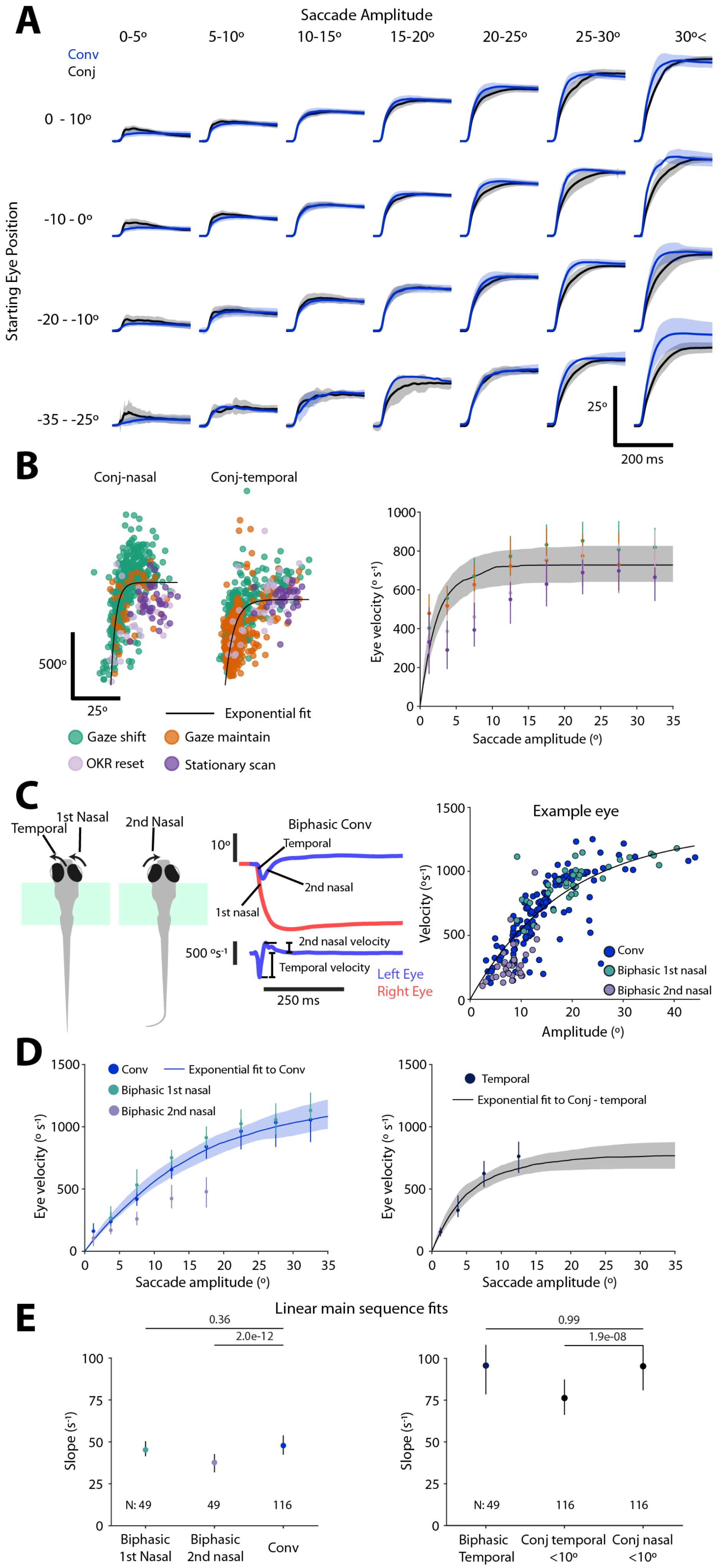

A
Saccade Amplitude
0-5° 5-10° 10-15° 15-20° 20-25° 25-30° 30°<
Conv
Conj
Starting Eye Position
0 - 10°
-10 - 0°
-20 - -10°
-35 - -25°
25°
200 ms
B
Conj-nasal
Conj-temporal
500°
25°
Exponential fit
Gaze shift
Gaze maintain
OKR reset
Stationary scan
Eye velocity (° s-1)
Saccade amplitude (°)
C
Temporal
1st Nasal
2nd Nasal
Biphasic Conv
10°
Temporal
2nd nasal
1st nasal
500 °s-1
2nd nasal velocity
Temporal velocity
250 ms
Left Eye
Right Eye
Example eye
Velocity (°s-1)
Amplitude (°)
Conv
Biphasic 1st nasal
Biphasic 2nd nasal
D
Conv
Exponential fit to Conv
Biphasic 1st nasal
Biphasic 2nd nasal
Eye velocity (° s-1)
Saccade amplitude (°)
Temporal
Exponential fit to Conj - temporal
E
Linear main sequence fits
0.36
2.0e-12
Slope (s-1)
N: 49
49
116
Biphasic 1st Nasal
Biphasic 2nd nasal
Conv
0.99
1.9e-08
N: 49
116
116
Biphasic Temporal
Conj temporal <10°
Conj nasal <10°

Figure S5: Velocity main sequence relationships – additional data. Related to Figure 6.

(A) Eye position time series for adducting saccades binned by amplitude and pre-saccadic eye position. (B) Velocity main sequence for sub-types of conjugate saccade. *Left:* An example eye with exponential fit and colour-coded sub-types of conjugate saccade. *Right:* Average velocity main sequence fit for conjugate saccades (reproduced from Figure 6D), overlaid with velocity data for each subtype of conjugate saccade (median and IQR per amplitude bin). (C) *Left:* Illustration of biphasic convergent saccade, with component eye movements indicated. *Middle:* Position and velocity time series from an example biphasic convergent saccade. *Right:* Example eye showing exponential fit to regular convergent saccade data as well as components of biphasic saccades. (D) *Left:* Average velocity main sequence for regular convergent saccades (reproduced from Figure 6D), overlaid with velocity data for biphasic convergent saccades. *Right:* Average velocity main sequence for temporal eye movements within conjugate saccades, overlaid with velocity data for temporal components of biphasic convergent saccades. (E) Linear velocity main sequence fits. *Left:* Convergent saccades and nasal components of biphasic saccades. *Right:* Small amplitude ($\leq 10°$) conjugate saccades and temporal component of biphasic saccades. Linear fits made for eyes with at least 10 saccades (n eyes indicated). Fit slope coefficients plotted as median (IQR). p-values from Kruskal-Wallis with Dunn-Sidak post-hoc tests.
